# The effect of alcohol cue exposure and acute intoxication on inhibitory control processes and ad libitum alcohol consumption

**DOI:** 10.1007/s00213-019-05212-4

**Published:** 2019-03-27

**Authors:** Laura Baines, Matt Field, Paul Christiansen, Andrew Jones

**Affiliations:** 1 Department of Psychological Sciences, University of Liverpool, L69 7ZA, Liverpool, UK; 2 UK Centre for Tobacco and Alcohol Studies, University of Liverpool, Liverpool, UK; 3 Department of Psychology, University of Sheffield, Sheffield, UK

**Keywords:** Alcohol, Craving, Cue reactivity, Inhibitory control, Proactive slowing, Signal detection, Stop-signal task

## Abstract

**Rationale:**

Alcohol intoxication and alcohol cue exposure impair ‘reactive’ inhibitory control and increase motivation to drink. However, inhibitory control is a multi-component process that also comprises signal detection and proactive control. It is unknown whether intoxication and cue exposure selectively influence these subprocesses in heavy drinkers.

**Objectives:**

In two pre-registered studies, we investigated whether exposure to alcohol-related cues (study 1) and alcohol priming (study 2) impair each of these subprocesses of inhibitory control and increase motivation to drink.

**Methods:**

In study 1, 64 heavy drinkers completed a modified stop-signal task in an alcohol context (with embedded alcohol cues) and a neutral context (with embedded neutral cues) followed by a subjective measure of craving and a bogus taste test to measure ad libitum alcohol consumption. In study 2, 36 heavy drinkers consumed an alcoholic beverage (0.6 g/kg body weight), an alcohol-placebo beverage, and water on a within-subjects basis, followed by the modified stop-signal task and a bogus taste test.

**Results:**

In study 1, alcohol cue exposure did not impair inhibitory control subprocesses. Reactive control was unexpectedly better following alcohol cue exposure (compared to neutral cue exposure). However, craving and ad libitum consumption increased as expected. In study 2, reactive control was significantly impaired following the alcohol and control primes, relative to the placebo, but there was no effect on proactive slowing or signal detection. As expected, intoxication increased motivation to drink and ad libitum consumption (compared to placebo and control).

**Conclusions:**

Alcohol intoxication and cue exposure increase motivation to drink in the absence of impairments in subcomponents of inhibitory control.

**Electronic supplementary material:**

The online version of this article (10.1007/s00213-019-05212-4) contains supplementary material, which is available to authorized users.

## Introduction

Inhibitory control is defined as the (in)ability to suppress, postpone or alter a response that is no longer appropriate (Logan et al. 1984) and can be measured using the stop signal and go/no-go computerised tasks. These tasks require the inhibition of a pre-potent motor response following a ‘stop signal’ or ‘no-go’ cue, and provide an index of inhibitory failures (commission errors) or latency to inhibit (stop-signal reaction time; SSRT). Theoretical models of addiction suggest a failure or impairment in inhibitory control is a candidate psychological mechanism for the development and maintenance of substance misuse (e.g. de Wit 2009; Fillmore 2003; Goldstein and Volkow 2002; Yucel et al. 2018). These predictions are supported by empirical evidence indicating that impairments in inhibitory control predict variance in hazardous drinking (Colder and O'Connor 2002; Houston et al. 2014), and meta-analyses demonstrating that inhibition is impaired in heavy drinkers and substance-dependent patients compared to controls (Smith et al. 2014). Longitudinal studies have also demonstrated that impaired inhibitory control predicts the onset of alcohol-related problems in at-risk adolescents (Nigg et al. 2006), the transition from heavy drinking to alcohol dependence (Rubio et al. 2008) and treatment success (Rupp et al. 2016).

Whilst the association between inhibitory control and alcohol (mis)use is seemingly well established, several ‘null’ findings have also been published (e.g. Kamarajan et al. 2005; Nederkoorn et al. 2009) and on closer inspection inhibitory control may only explain a modest amount of variance in substance use behaviour (Smith et al. 2014). One potential explanation for this is a *simplistic* conceptualization of inhibitory control. Cognitive neuroscience models (Verbruggen et al. 2014a) emphasise the importance of the underlying mechanistic processes that contribute to engagement of inhibitory control. For example, SSRT—the estimated time to withhold a response following the presentation of a stop signal (Brevers et al. 2017)—is regularly used as an index of inhibitory control. However, SSRT represents more than simply the time taken to inhibit a response, because effective stopping relies on initial detection of the stop signal (‘signal detection’), the selection of an appropriate response (‘response selection’), followed finally by execution of the stopping response. Importantly, Verbruggen et al. (2014b) demonstrated that signal detection contributed to the response inhibition process and can be isolated in stop-signal tasks through calculating differences in SSRTs on blocks when the stop signal is presented in the centre of the screen, compared to blocks when the stop signal is presented in the periphery. Additionally, although reactive control (SSRT; the act of stopping) is an important aspect of executive control and has been the focus of most research in substance use, we also have the ability to plan our behaviour and alter this ‘proactively’ (Verbruggen et al. 2014a). This preparatory response has a downstream impact on ‘reactive stopping.’ Proactive slowing can be inferred by examining the difference in reaction times in blocks where inhibitory signals are present and blocks where these signals are absent and no inhibition is required (Aron 2011). Indeed, research has shown that participants employ proactive adjustments in order to ready themselves to detect a stop signal and, therefore, slow down their responses (Elchlepp et al. 2016; Verbruggen and Logan 2009b; Zandbelt et al. 2011). Although these additions may increase task difficulty, we can investigate whether these additional processes influence performance on stop-signal tasks and if reactive control alone is limited as a model of executive control (Aron 2011).

Importantly, both signal detection and proactive control may have a significant role in substance use behaviour (Brevers et al. 2017). First, substance users’ selective attention is guided by substance-related cues (Townshend and Duka 2001) and impaired by alcohol (Plawecki et al. 2018; Roberts et al. 2014), which may make it difficult to detect inhibitory signals in the environment. Second, substance users rarely engage global reactive stopping responses in the real world (i.e. reaching for a glass but then inhibiting), but regularly engage proactive control processes (i.e. preparation in advance, such as declining to order an alcoholic drink). Therefore, to better understand the association between inhibitory control and alcohol use, we need to account for the influence of preparation and signal detection on inhibitory control (Verbruggen et al. 2014a).

A second issue which may impact the association between inhibitory control and alcohol use is the stability of the processes. The majority of research suggests inhibitory control is stable over long periods. However, more recent evidence suggests inhibitory control may fluctuate over time *within* individuals, suggesting that the capacity to proactively prepare, choose and stop a response is fluid. In a narrative review (Jones et al. 2013), we identified various situational and internal triggers, for example, alcohol-related cues, alcohol intoxication, ego depletion and stress, which may cause short-term deficits in inhibitory control (see also de Wit 2009). Subsequent empirical research has demonstrated limited evidence for stress-related impairments in inhibitory control (Scholz et al. 2009) and the veracity of the ego-depletion effect is under debate (Hagger et al. 2016). Nevertheless, the effects of acute intoxication and cue exposure on inhibitory control are seemingly robust, with a systematic review (Weafer and Fillmore 2016) demonstrating alcohol intoxication consistently impairs inhibitory control and recent meta-analyses demonstrating small but robust effects of alcohol cue exposure on inhibitory control (Jones et al. 2018).

Across the majority of studies included in these evidence syntheses, the focus was global reactive control indices (SSRTs or No/Go errors), and currently, there is little research investigating the effects of alcohol cues and intoxication on inhibitory subprocesses (specifically, proactive slowing and signal detection). In one study, Sharma (2017) showed how preceding alcohol cues (compared to neutral cues) impaired the performance of heavy drinkers, but not light drinkers, on a modified Stroop task. These results implied that heavy drinkers were relying on reactive control, whereas light drinkers were employing proactive control to filter out the context of the prior image. Conversely, Campbell et al. (2017) demonstrated that alcohol intoxication increased motor SSRTs but did not influence proactive slowing. Indeed, this emphasises the simplistic conceptualization of inhibitory control in the majority of prior research and the need to break inhibitory control down into its component processes to further understanding.

Consequently, the current studies aimed to directly investigate the effect of alcohol cue exposure (study 1) and alcohol intoxication (study 2) on the different components of inhibitory control (namely reactive stopping, signal detection and proactive control), and subsequent craving and ad libitum alcohol consumption. We included these alcohol-seeking measures due to substantial evidence demonstrating that both alcohol-related cues (Fatseas et al. 2015; MacKillop and Lisman 2007) and alcohol intoxication (Christiansen et al. 2012; De Wit and Chutuape 1993) increase motivation to consume subsequent alcohol. We also aimed to investigate whether increased alcohol seeking was the product of impairments in the different components of control as past research has demonstrated that impairments in inhibitory control predict hazardous drinking (Colder and O'Connor 2002; Houston et al. 2014). We pre-registered the design, statistical power calculations, hypotheses and analysis strategy, with data freely available on Open Science Framework (study 1: [https://osf.io/qf72a/], study 2: [https://osf.io/dg27x/]).

## Study 1

We hypothesised that exposure to alcohol-related cues compared to neutral cues would (i) impair reactive control, signal detection and proactive slowing and (ii) increase self-reported craving and subsequent ad libitum alcohol consumption. We also hypothesised that (iii) deficits in proactive slowing and signal detection would predict unique variance in alcohol consumption after controlling for reactive inhibition. Finally, we hypothesised that (iv) the effects of alcohol cue exposure on ad libitum alcohol consumption would be partially mediated by changes in the different components of control.

## Methods

### Participants

Heavy drinkers (*N* = 64; 37 females, 27 males) took part in a laboratory study across two sessions, approximately one week apart. Participants were aged between 18 and 59 (M = 23.73, SD = 9.33) and were recruited from the University of Liverpool and wider community through online advertisements. We conducted a power analysis to detect a within × between interaction (*d* = .39, *α* = .05, 1 − *β* = 90%) based on a pooled effect size from studies which have examined the effect of alcohol-related cues on inhibitory control in heavy drinkers (e.g. Czapla et al. 2015; Kreusch et al. 2013). Heavy drinking was defined using UK government guidelines: males and females who consume > 14 UK units of alcohol per week (1 UK unit = 8 g of pure alcohol). Eligibility criteria included age 18 or over, a fluent English speaker and a self-reported motivation to reduce their alcohol consumption. We recruited individuals who reported motivation to restrict consumption as these individuals should be employing inhibitory control to restrict their intake (Hofmann et al. 2012). Exclusion criteria included self-reported current or previous diagnosis of substance use disorder, ADHD, psychiatric disorder, a current/recent illness (e.g. flu) that could increase sensitivity to alcohol, taking medications (e.g. antidepressants) that are adversely affected by alcohol, pregnancy or breastfeeding. The study was approved by the University of Liverpool’s local ethics committee.

### Materials

#### Questionnaires

Participants completed a battery of questionnaires; this included a two-week timeline follow back (TLFB: Sobell and Sobell 1990) to measure retrospective alcohol consumption in units, the Alcohol Use Disorders Identification Test (AUDIT: Saunders et al. 1993) to measure hazardous drinking (study 1: *α* = .66, study 2: *α* = .66), the Brief Comprehensive Effects of Alcohol Questionnaire (B-CEAQ: Ham et al. 2005) to measure alcohol outcome expectancies (study 1: *α* = .84 study 2: *α* = .80), the Temptation Restraint Inventory (TRI: Collins and Lapp 1992) to measure drinking restraint (preoccupation with and efforts to reduce drinking) (study 1: *α*’s > .61, study 2: *α*’s > .54) and the Barratt Impulsivity Scale (BIS: Patton et al. 1995) to measure self-reported impulsivity across three dimensions (motor, non-planning and attentional) (study 1: *α*’s > .61, study 2: *α*’s > .44).

To measure self-reported craving before and after the stop-signal task, participants completed the Approach and Avoidance of Alcohol Questionnaire ‘right now’ version (AAAQ: McEvoy et al. 2004) which consists of three subscales of craving (inclined/indulgent, obsessed/compelled, resolved/regulated) (study 1: *α*’s > .64, study 2: *α*’s > .78). Participants also completed a funnelled debrief to measure awareness of the experimental aims of the study. This included an open question asking what the purpose of the experiment was and two fixed-response questions asking the purpose of the computer task and the taste test (see supplementary materials).

#### Modified stop-signal task (SST; Verbruggen et al. 2014b)

Participants completed a modified stop-signal task, designed to isolate proactive slowing, reactive control and signal detection. At the beginning of each trial, a white fixation line appeared in the middle of the screen for 500 ms, as well as a white border around the edge of the screen display. Following these, two words appeared, one immediately above the line and one immediately below the fixation line. These words described natural (e.g. lion, swan) or man-made (e.g. desk, shed) objects, based on Verbruggen et al. (2014b). Natural words were assigned as targets, and participants had to respond as quickly as possible to their position in relation to the line (above or below) by a key press. Man-made words were distractors. Depending on condition, a neutral-related image (e.g. a scene from an office) or alcohol-related image (e.g. a scene from a bar) appeared in the background on each trial. There were 10 of each image type, and they were 230 mm × 130 mm in size. The task consisted of three blocks (no-signal block, central-signal block, peripheral-signal block), which were presented in a randomised, counterbalanced order.

##### No-signal block

In this block, participants had to identify the position of the target word in relation to the line without interruption on 100% of trials (128 in total).

##### Central-signal block

In this block, participants had to identify the position of the target word in relation to the line without interruption on 75% of trials (96 in total). The remaining 25% of trials (32 in total) were stop-signal trials, in which the white fixation line between the words increased in size by 300%. Participants were told to try and withhold their response to the target word position if this happened.

##### Peripheral-signal block

In this block, participants identified the position of the target word in relation to the line without interruption on 75% of trials (96 in total). The remaining 25% of trials (32 in total) were stop-signal trials, in which the white square around the edge of the display increased in size by 300%. Participants were told to try and withhold their response to the target word position if this happened.

Participants were also given standard stop-signal instructions in which they were explicitly told that they should not wait for the signal and should, instead, respond as quickly as possible. In both the central-signal and peripheral-signal blocks, the delay between presentation of the target and distractor word and the increase in size of the stop signals (fixation line or square around the display) was adjusted on a trial-by-trial basis using a tracking procedure (Verbruggen and Logan 2009a). In each block, the initial delay was 250 ms; if participants failed to inhibit, the delay decreased by 50 ms, making subsequent inhibition easier; if participants successfully inhibited, then the delay increased by 50 ms, making subsequent inhibition more difficult.

In line with our pre-registration, reactive control was inferred as the mean SSRT (Verbruggen et al. 2013) collapsed across central- and peripheral-signal blocks. However, we also examined SSRTs based only on central-signal blocks in order to provide a more direct comparison with previous literature. Proactive slowing was inferred from the degree of reaction time slowing on both stop-signal blocks compared to no-signal blocks (RTstop signal − RTno signal). Signal detection was inferred from the difference in SSRT (SSRTperipheral signal − SSRTcentral signal) between central-signal and peripheral-signal blocks. The effects of alcohol cues on each process were measured by comparing performance across conditions (alcohol context, neutral context).

#### Ad libitum taste test

Participants received 250 ml of chilled Skol beer (2.8% vol. ABV) and 250 ml of chilled fresh orange juice (non-alcoholic beverage). They were not informed of the brands used and were given each drink simultaneously in unmarked glasses. Participants were asked to taste and rate the drinks on various gustatory dimensions e.g. ‘*How bitter did you find the drink?*’ using visual analogue scales and were told to ‘*drink as much or as little as you like in order to make accurate judgements*’. Before completion, participants were also told that alcohol would impair performance on the next task, in which they had the opportunity to win small amounts of money, in order to increase their motivation to restrict their intake (taken from Christiansen et al. 2012; Field and Jones 2017). The volume of each drink consumed was recorded unobtrusively at the end of each session, and ad libitum alcohol consumption was expressed as the amount of beer as a percentage of total fluid consumed.

### Procedure

Participants attended two sessions approximately one week apart, the order of which was counterbalanced. One session was completed in a standard neutral laboratory; the other was completed in the University of Liverpool’s Bar Laboratory (https://www.liverpool.ac.uk/psychology-health-and-society/departments/psychological-sciences/facilities/bar-lab/) which resembles a typical UK bar containing advertisements for alcohol, beer pumps, etc. Participants were breathalysed at the beginning of each session and were required to have a breath alcohol concentration (BAC) of 0.0 mg/l in order to take part. Participants first provided demographic information and completed the battery of questionnaires measuring alcohol use and personality (TLFB, AUDIT, B-CEAQ, TRI and BIS) and the AAAQ to measure craving before the SST. Before each block of the task, participants were asked to smell a drink and allow a small amount to touch their lips (beer in the alcohol session, water in the neutral session), to increase cue reactivity further (see Field and Jones 2017). Following the SST, participants completed a second AAAQ to measure craving following the task. They then completed the taste test followed by a Balloon Analogue Risk task (BART; Lejuez et al. 2003). During this task, participants had to click a mouse to pump up 10 simulated balloons. Each pump was worth £0.05 which they could collect in a ‘permanent bank’. However, if the balloon burst before collection, participants lost the money from that trial. This task was presented to ensure participants believed our cover story, that alcohol might impair their performance. Our hypotheses did not concern performance on this task, and as a result, it is not reported here (see supplementary materials for further details). Participants then provided a final breath alcohol sample, and in the final session completed a funnelled debrief assessing awareness of experimental measures (see supplementary analyses).

### Data reduction and analysis

For the stop-signal task, outliers were removed following criteria suggested in previous research (Field and Jones 2017; Verbruggen and De Houwer 2007). Reaction times that were greater than 2000 ms or less than 100 ms were removed, as were reaction times that were 2.5 standard deviations greater or less than individual means. We also checked for outliers during examination of box-and-whisker plots.^1^ Two participants were removed from the stop-signal task analysis as the data did not record for one block. One participant did not complete the taste test during the neutral session as they stated they had not eaten during the day of testing. Details of how each hypothesis was analysed are included in the pre-registration. Post hoc comparisons were carried out using LSD tests.

### Results

#### Sample characteristics

Participants consumed 53.64 (± 35.64) units on average in the two weeks prior to their participation in the study, and reported an average AUDIT score of 12.59 (± 4.65), indicative of hazardous drinking. An independent *t* test revealed no significant differences in AUDIT scores between males (13.48 ± 5.21) and females (11.95 ± 4.16; *t* (62) = 1.31, *p* = .195, *d* = 0.33). However, males consumed significantly more units (68.87 ± 46.16) in the two weeks prior to the study compared to females (42.53 ± 19.56; *t* (33) = 2.79, *p* = .009, *d* = 0.71).

#### Hypothesis 1: Does alcohol cue exposure cause deficits in inhibitory processes? (See Table 1)

Deficits in signal detection and reactive control were analysed using a 2 (block: central signal, peripheral signal) × 2 (condition: alcohol cue exposure, neutral cue exposure) repeated-measures ANOVA on SSRTs. This revealed a significant main effect of block (*F* (1, 61) = 36.99, *p* < .001, *η*_*p*_^2^ = .38) where SSRTs were significantly faster for central compared to peripheral blocks. This indicates greater reactive stopping when the stop signal was presented centrally compared to in the periphery. There was also a main effect of condition (*F* (1, 61) = 4.52, *p* = .038, *η*_*p*_^2^ = .07), but contradictory to our hypothesis, SSRTs were significantly faster (indicating better reactive stopping) during alcohol cue exposure compared to neutral cue exposure. Furthermore, there was no interaction between block and condition (*F* (1, 61) = 3.02, *p* = .087, *η*_*p*_^2^ = .05) suggesting that cue exposure did not impair signal detection. We also compared SSRTs in central stop-signal blocks only, and this revealed no significant differences in SSRTs following alcohol cue exposure compared to neutral cue exposure (*t* (61) = − .74, *p* = .463, *d* = − 0.11), again suggesting that alcohol cues did not impair reactive control.

**Table 1:** Descriptive statistics for SSRTs and mean go-reaction times (ms) shown separately for each condition (values are mean, SD)

	Alcohol cue exposure	Neutral cue exposure
SSRT (central)	426.13 (108.39)	437.32 (102.34)
SSRT (peripheral)	475.48 (132.71)	526.12 (156.64)
Overall SSRT	450.81 (103.27)	481.72 (116.30)
No-signal block RT	714.75 (101.78)	757.15 (114.72)
Signal block RT (central)	946.11 (233.52)	963.67 (182.66)
Signal block RT (peripheral)	945.29 (229.26)	971.08 (168.05)

Lower score = faster SSRT. Overall SSRT = mean of the peripheral and central SSRTs

Proactive slowing was analysed using a 2 (block: no-signal block, central- and peripheral-signal blocks) × 2 (condition: alcohol cue exposure, neutral cue exposure) repeated-measures ANOVA on reaction times. This showed a main effect of block (*F* (1, 61) = 134.47, *p* < .001, *η*_*p*_^2^ = .69) whereby participants slowed down their responses more in the stop-signal blocks compared to the no-signal blocks indicative of proactive slowing. Furthermore, there was a main effect of condition (*F* (1, 61) = 5.34, *p* = .024, *η*_*p*_^2^ = .08) whereby participants were slower to respond during neutral cue exposure compared to alcohol cue exposure. However, there was no significant interaction between block and condition (*F* (1, 61) = 1.11, *p* = .295, *η*_*p*_^2^ = .02) suggesting that alcohol cue exposure did not impair proactive slowing.

#### Hypothesis 2: Does alcohol cue exposure increase craving and ad libitum alcohol consumption? (See Table 2)

To examine whether alcohol cue exposure increased craving, scores on the AAAQ were analysed using a 3 (subscale: mean scores on inclined/indulgent, obsessed/compelled, resolved/regulated) × 2 (time: pre-manipulation, post-manipulation) × 2 (condition: alcohol cue exposure, neutral cue exposure) repeated-measures ANOVA. This revealed that there was no main effect of condition (*F* (1, 63) = 1.31, *p* = .257, *η*_*p*_^2^ = .02) or time (*F* (1, 63) = 2.41, *p* = .125, *η*_*p*_^2^ = .04). However, there were significant condition × time (*F* (1, 63) = 11.96, *p* = .001, *η*_*p*_^2^ = .16) and condition × time × AAAQ subscale (*F* (2, 114) = 5.95, *p* = .005, *η*_*p*_^2^ = .09) interactions.

**Table 2:** AAAQ scores before and after the modified stop-signal task split by experimental condition (values are mean, SD)

	Alcohol cue exposure		Neutral cue exposure	
	Pre-task	Post-task	Pre-task	Post-task
Inclined/indulgent	4.61 (1.54)	4.74 (1.58)	5.05 (1.44)	4.59 (1.68)
Obsessed/compelled	0.75 (0.89)	0.95 (1.05)	0.91 (1.04)	0.88 (1.03)
Resolved/regulated	1.28 (1.14)	1.15 (1.22)	1.38 (1.22)	1.38 (1.22)

To examine these interactions further, a 2 × 2 ANOVA was conducted on each subscale separately. For the *inclined/indulgent* subscale, there was no main effect of condition (*F* (1, 63) = 0.79, *p* = .378, *η*_*p*_^2^ = .01). However, there was a main effect of time (*F* (1, 63) = 4.15, *p* = .046, *η*_*p*_^2^ = .06), with scores decreasing post-manipulation. There was also a significant condition × time interaction (*F* (1, 63) = 13.45, *p* = .001, *η*_*p*_^2^ = .18). This revealed a decrease from pre- to post-manipulation following neutral cue exposure (*p* < .001) but no difference between pre- and post-manipulation following alcohol cue exposure (*p* = .279). This suggests craving did not significantly increase following alcohol cue exposure. Lastly, there was no difference at post-manipulation between the two conditions (*p* = .437). For the *obsessed/compelled* subscale, there was a condition × time interaction (*F* (1, 63) = 6.82, *p* = .011, *η*_*p*_^2^ = .10) demonstrating that participants reported greater craving post-manipulation compared to pre-manipulation following alcohol cue exposure (*p* = .025) but no difference following neutral cue exposure (*p* = .768). There was also no difference between the conditions at post-manipulation (*p* = .524). Lastly, there was only a main effect of time on the *resolved/regulated* scale (*F* (1, 63) = 6.21, *p* = .015, *η*_*p*_^2^ = .09) which showed scores decreased at post-manipulation in both conditions.

**Fig. 1:**
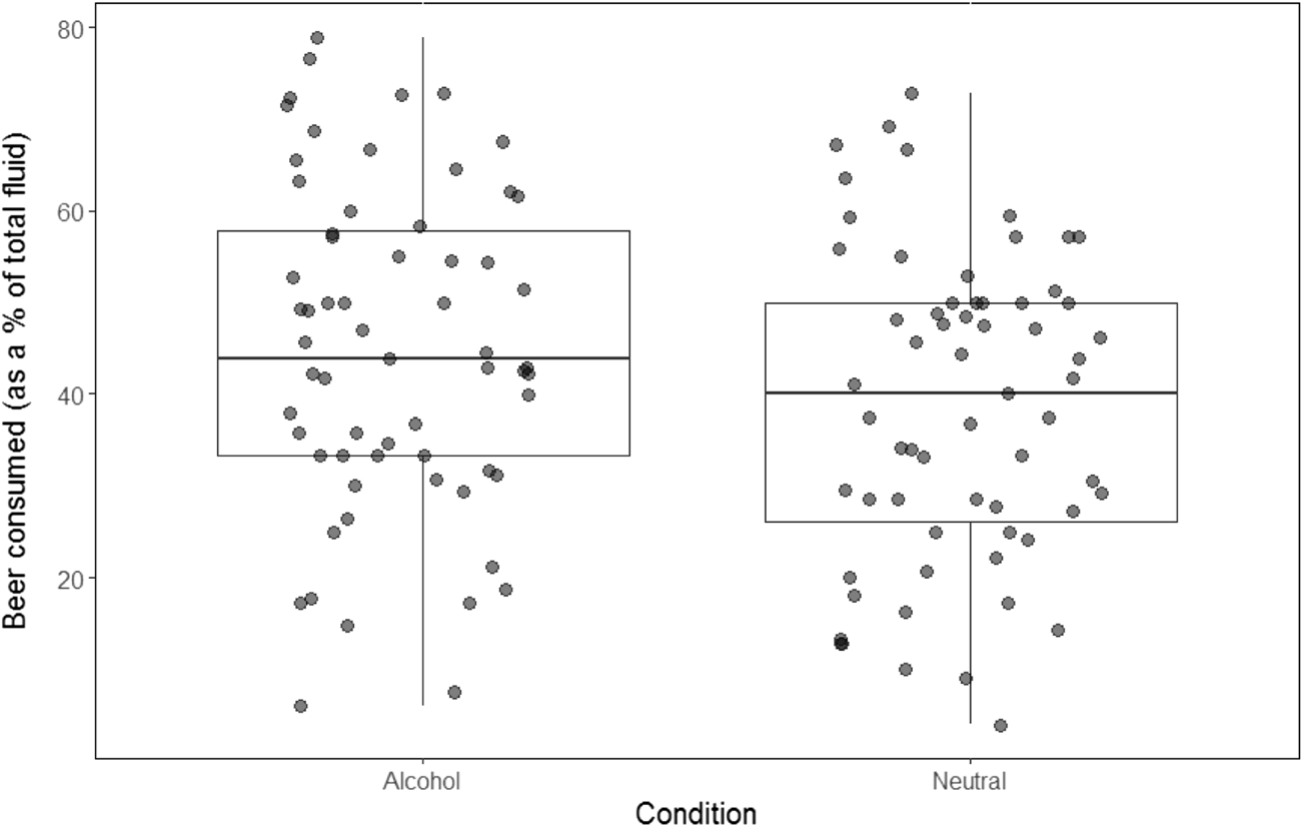
Boxplot to show beer consumed as a percentage of total fluid following alcohol cue exposure and neutral cue exposure (*N* = 63)

To examine differences in ad libitum alcohol consumption, we conducted paired-samples *t* tests on beer consumed (as a percentage of total fluid). This revealed that participants drank significantly more beer following alcohol cue exposure compared to neutral cue exposure (*t* (62) = 2.66, *p* = .01, *d* = 0.34; see Fig. 1). Finally, there was no significant difference in ratings of alcohol pleasantness following alcohol cue exposure (6.33 ± 2.31) compared to neutral cue exposure (6.11 ± 2.13; *t* (62) = 0.96, *p* = .34, *d* = 0.12) (see supplementary materials for further details).

We also hypothesised that deficits in proactive slowing and signal detection would predict unique variance in alcohol consumption after controlling for reactive inhibition, and that the effects of alcohol cues on ad libitum alcohol consumption would be partially mediated by changes in the different components of control. However, we did not demonstrate impairments due to alcohol cue exposure and deficits in inhibitory control did not predict alcohol consumption. Hence, we do not meet the assumptions required to examine within-subjects mediation (see supplementary materials).

### Interim discussion

Study 1 demonstrates that alcohol cue exposure did not impair inhibitory subprocesses. Indeed, reactive control was unexpectedly better following alcohol cue exposure (compared to neutral cue exposure) when examining central and peripheral stop-signal blocks, although there was no difference when analysing central blocks only. Furthermore, although there was the presence of proactive slowing and increased signal detection of central stop signals (compared to periphery), neither proactive slowing nor signal detection were directly impaired by alcohol cues. In line with previous research, alcohol cue exposure increased craving (albeit weakly) and subsequent ad libitum alcohol consumption. However, this was not the result of impairments in inhibitory subprocesses.

## Study 2

In study 2, we administered a control, placebo-alcohol and alcohol prime to investigate the pharmacological and anticipated effects of alcohol on inhibitory subprocesses and motivation to drink. Typical alcohol priming studies compare the effects of an alcohol dose and a placebo dose to investigate the pharmacological effects of alcohol (e.g. Fillmore et al. 2009; Marczinski et al. 2005; Weafer and Fillmore 2008). However, this comparison has low ecological validity as in the real world it is likely that the effect of alcohol is the result of both the pharmacological and the anticipated effects. Therefore, with the addition of a control condition, we are able to distinguish the anticipated from the pharmacological effects of alcohol (Christiansen et al. 2012).

We hypothesised that acute alcohol intoxication compared to placebo and control would (i) cause deficits in reactive control, signal detection and proactive slowing and (ii) increase alcohol-seeking measures.^2^ We also hypothesised that (iii) following alcohol intoxication, proactive slowing, signal detection and reactive control would predict unique variance in alcohol consumption. Finally, we hypothesised that (iv) the effects of alcohol intoxication on ad libitum alcohol consumption would be partially mediated by changes in the different components of control.

## Methods

### Participants

Heavy drinkers (*N* = 36; 19 males) took part in a laboratory study with three sessions, approximately one week apart. Participants were aged between 18 and 44 (M = 24.75, SD = ± 7.33). The number of participants was decided upon using a power calculation to find a medium effect size (*d* = .50) at *α* = .05, and 90% power. Studies have demonstrated larger effect sizes of alcohol impairments on inhibitory control (Stroop) tasks (e.g. Rose and Duka 2007, *d* = .89); however, as no research has examined the effects on inhibitory subcomponents, we opted for a more conservative estimate of *d* = .50. Inclusion and exclusion criteria and recruitment strategy were the same as those of study 1.

### Materials

#### Questionnaires

Participants completed the same questionnaires and awareness of experimental aims questions (see supplementary materials) that are described in the method of study 1. They also completed the *Subjective intoxication scales* (SIS; Duka et al. 1998) to measure subjective feelings of ‘lightheaded’, ‘irritable’, ‘stimulated’, ‘alert’, ‘relaxed’ and ‘contented’ following alcohol priming. We also asked participants how many alcohol units they believed they had consumed in the priming drink in each session.

#### Stop-signal task (SST; Verbruggen et al. 2014b)

Participants completed a modified stop-signal task, which was nearly identical to task 1, the only difference being that we removed the alcohol and neutral-related images in order to prevent contamination of findings with cue exposure. Therefore, the task was presented on a black background across each block and session.

#### Procedure

Participants attended three sessions (alcohol, placebo and control) in a neutral laboratory. Each session took place between 12 p.m. and 6 p.m. and had to be at least one week apart. The sessions were completed in a pseudo-counterbalanced order. In line with previous studies, participants completed the control session first, followed by either the placebo or alcohol session in a counterbalanced order. Participants were informed that the experiment was investigating the effect of a high, low and no dose of alcohol on taste perception. Participants were breathalysed at the beginning of each session, and a BAC of 0.0 mg/l was required in order to take part.

Participants first completed the demographic questions and a battery of questionnaires measuring personality and alcohol use (first session only). They then completed the AAAQ and dependent on condition, received either the alcohol, placebo or control drink (in two glasses) and were asked to consume this within 10 min, followed by a 20-min absorption period.

The alcoholic drink contained vodka (Smirnoff Red, 37.5% alcohol by volume (ABV)) and chilled tonic water. The alcohol dose was calculated as 0.6 g/kg of body weight (maximum dose of 200 ml vodka/8 UK units) and the drink mixed one part vodka, three parts tonic water. The placebo-alcohol drink contained chilled tonic water, the total volume of which was the same as that of the alcoholic drink. Vodka mist was sprayed onto the surface of the drink and smeared onto the rim of the glass to simulate the smell and taste of alcohol. Tabasco sauce was also added to the drink to give the burning sensation of alcohol. The control drink consisted of chilled water; the total volume was identical to the alcoholic and placebo drink. This procedure is similar to previous research carried out (e.g. Christiansen et al. 2012).

Participants then completed the AAAQ and SIS, and provided a breath alcohol sample, before completing the SST. Following the SST, participants completed the ad libitum taste test (see study 1 method) and were informed that alcohol may impair their performance on the last task, in which they had the opportunity to win small amounts of money. Lastly, participants completed the BART task (see study 1 procedure/supplementary materials) and provided a final breath alcohol sample.

#### Data analysis

SST data was handled using the same procedures as study 1. Two participants were excluded from the SST analysis due to outliers. One participant was removed from the analysis of the taste test as they did not complete this during one session. Further details on the analysis of each hypothesis can be found in the pre-registration.

### Results

#### Sample characteristics

Participants consumed an average of 48.90 (± 25.72) UK units in the two weeks prior to the first session of the study and reported a mean AUDIT score of 11.78 (± 4.81), indicative of hazardous drinking. There was no significant difference in AUDIT scores between males (11.32 ± 3.89) and females (12.29 ± 5.75; *t* (34) = − .60, *p* = .55, *d* = 0.20); however, males did consume significantly more units (60.32 ± 25.68) than females (36.15, ± 19.43; *t* (34) = 3.16, *p* = .003, *d* = 1.06) in the two weeks prior to taking part. There were no significant differences in drinking patterns of the participants across the two studies (see supplementary materials).

#### Hypothesis 1: Does alcohol intoxication cause deficits in inhibitory processes? (See Table 3)

Deficits in signal detection and reactive control were analysed using a 2 (block: central, periphery) × 3 (condition: control, alcohol, placebo) repeated-measures ANOVA on SSRTs. There was a significant main effect of block (*F* (1, 33) = 48.05, *p* < .001, *η*_*p*_^2^ = .59), with SSRTs significantly faster in the central stop-signal blocks compared to those of the peripheral stop-signal blocks. Similar to study 1, this indicates that reactive stopping was better when stop signals were presented centrally compared to in the periphery. There was also a main effect of condition (*F* (2, 66) = 3.44, *p* = .038, *η*_*p*_^2^ = .09) which revealed that as predicted SSRTs were significantly slower (indicating poorer reactive control) following alcohol intoxication compared to the placebo (*p* = .008). However, there was no difference following alcohol compared to the control prime (*p* = .841). Contrary to predictions, SSRTs were also significantly faster following the placebo compared to the control (*p* = .033) suggesting that the anticipated effects of alcohol did not impair reactive control. Lastly, there was no interaction between block and condition (*F* (2, 66) = 2.09, *p* = .132, *η*_*p*_^2^ = .06) indicating alcohol intoxication did not impair signal detection. For direct comparisons with previous research, we also investigated differences in SSRTs computed from central stop-signal blocks *only*. This also revealed a main effect of condition (*F* (2, 66) = 3.39, *p* = .04, *η*_*p*_^2^ = .09) which demonstrated that SSRTs were significantly slower following alcohol compared to a placebo (*p* = .018) but no difference following alcohol compared to a control (*p* = .084). However, there was also no difference between control compared to the placebo primes (*p* = .449), again demonstrating there was no anticipated impairing effects of alcohol on reactive control.

**Table 3:** Descriptive statistics for SSRTs and mean go-reaction times (ms) shown separately for each condition (values are mean, SD)

	Control	Alcohol	Placebo
SSRT (central)	378.39 (76.26)	410.39 (81.39)	364.86 (84.59)
SSRT (periphery)	512.11 (176.87)	490.48 (174.51)	431.50 (105.64)
Overall SSRT	445.25 (109.54)	450.44 (109.69)	398.18 (85.58)
No-signal block RT	708.67 (90.77)	670.85 (77.59)	691.27 (113.87)
Signal block RT (central)	948.71 (180.38)	887.37 (187.88)	879.85 (192.15)
Signal block RT (periphery)	976.68 (170.86)	894.70 (218.66)	940.19 (206.74)

Lower score = faster SSRT. Overall SSRT = mean of the periphery and central SSRTs

Deficits in proactive slowing were analysed using a 2 (block: no signal, stop signal) × 3 (condition: control, alcohol, placebo) repeated-measures ANOVA on mean go-reaction times. In line with study 1, this revealed a significant main effect of block (*F* (1, 33) = 81.13, *p* < .001, *η*_*p*_^2^ = .71). Participants responded significantly faster in the no-signal block compared to the stop-signal blocks indicating the presence of proactive slowing. There was also a main effect of condition (*F* (2, 66) = 3.64, *p* = .032, *η*_*p*_^2^ = .10) which revealed that participants were slower to respond in the control session compared to the alcohol (*p* = .011). However, there was no difference following the alcohol prime compared to the placebo (*p* = .292) or following the placebo compared to the control (*p* = .132). Most importantly, there was no interaction between block and condition (*F* (2, 66) = 0.89, *p* = .415, *η*_*p*_^2^ = .03) suggesting that alcohol intoxication did not impair proactive slowing.

#### Hypothesis 2: Does alcohol intoxication increase alcohol seeking and consumption? (see Table 4)

Changes in craving subscales were assessed using a 3 (subscales: mean score on inclined/indulgent, obsessed/compelled and resolved/regulated) × 3 (condition: control, alcohol, placebo) × 2 (time: pre-drink, post-drink) repeated-measures ANOVA. There was no main effect of condition (*F* (2, 70) = 0.90, *p* = .41, *η*_*p*_^2^ = .03) or time (*F* (1, 35) = 2.54, *p* = .12, *η*_*p*_^2^ = .07). However, there was a significant condition × time interaction (*F* (2, 70) = 7.96, *p* = .001, *η*_*p*_^2^ = .19).

**Table 4:** Descriptive statistics for craving scores before and after the priming drinks (values are mean, SD)

	Inclined/indulgent	Obsessed/compelled	Resolved/regulated
Pre-control	5.12 (1.92)	1.22 (1.65)	1.33 (1.37)
Post-control	4.34 (2.36)	1.11 (1.59)	1.18 (1.33)
Pre-placebo	4.74 (1.89)	1.38 (1.87)	1.48 (1.45)
Post-placebo	4.27 (2.23)	1.41 (1.88)	1.08 (1.28)
Pre-alcohol	4.68 (1.67)	1.41 (1.80)	1.34 (1.47)
Post-alcohol	4.98 (2.11)	1.83 (2.04)	1.13 (1.50)

To examine the interaction, we conducted 3 (condition: control, alcohol, placebo) × 2 (time: pre-drink, post-drink) repeated-measures ANOVAs on each subscale individually. For both the *inclined/indulgent* and *obsessed/compelled* subscales, there was a significant condition × time interaction (inclined (*F* (2, 70) = 5.71, *p* = .005, *η*_*p*_^2^ = .14); obsessed (*F* (2, 70) = 3.98, *p* = .023, *η*_*p*_^2^ = .10)). The nature of these interactions demonstrated that participants reported lower scores on the inclined subscale at post-control compared to pre-control (*p* = .005) but there were no significant differences across time in the alcohol or placebo sessions (*p*s > .05). Across conditions, participants reported higher scores on the *inclined/indulgent* subscale following the alcohol prime compared to the placebo (*p* = .044) but there were no other significant differences between conditions. On the *obsessed/compelled* subscale, participants reported higher scores at post-drink in the alcohol session compared to pre-alcohol (*p* = .018) but there was no difference following the placebo or control drinks. Participants also reported higher scores following alcohol compared to the control (*p* = .004), but there were no other significant differences across conditions. For the *resolved/regulated* subscale, there was only a main effect of time (*F* (1, 35) = 10.90, *p* = .002, *η*_*p*_^2^ = .24) which demonstrated that participants felt less avoidant towards alcohol post-drinks compared to pre-drink. Notably, there were no significant differences in any of these measures pre-drink (*p*s > .05).

**Fig. 2:**
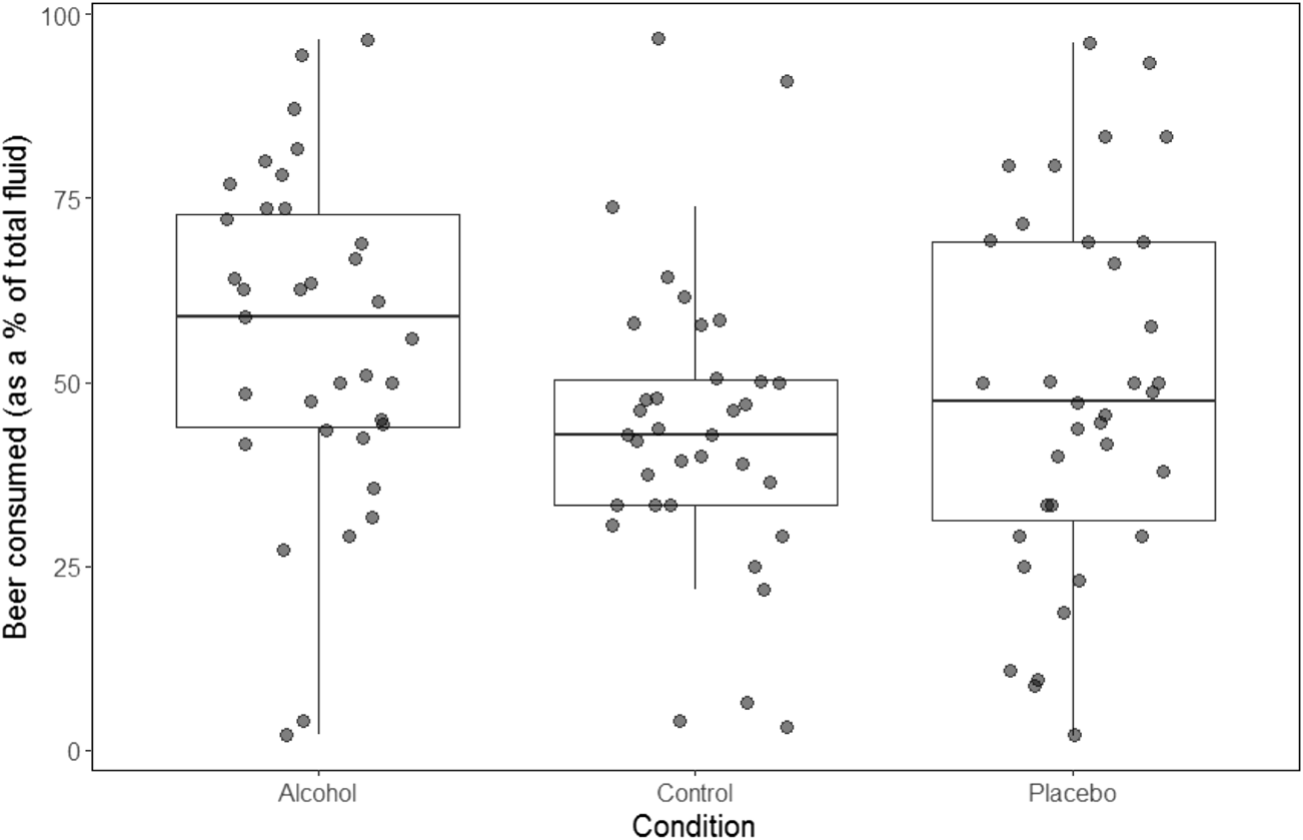
Boxplot of the mean consumption of beer (as a % of total fluid consumed) in the ad libitum taste test during the control, alcohol and placebo sessions (*N* = 35) (The removal of outliers from the control session did not significantly influence the comparison in beer consumption following the alcohol prime compared to the control, however the comparison following the alcohol prime compared to the placebo was no longer significant)

We also investigated if alcohol priming increased ad libitum alcohol consumption. There was a main effect of condition on beer consumed in the taste test (*F* (2, 68) = 5.98, *p* = .004, *η*_*p*_^2^ = .15). Participants drank significantly more beer following the alcohol prime compared to both control (*p* = .002) and placebo (*p* = .045) primes; however, there was no difference following the control compared to placebo prime (*p* = .199) (see Fig. 2). There was no main effect of condition on pleasantness ratings of beer (*F* (2, 68) = 1.89, *p* = .159, *η*_*p*_^2^ = .05).

For BACs, a 3 (condition: alcohol, placebo, control) × 2 (time: post-drink, end of session) repeated-measures ANOVA with 3 levels demonstrated a significant main effect of condition (*F* (1, 34) = 399.94, *p* < .001, *η*_*p*_^2^ = .92) with significantly higher BACs following the alcohol prime compared to the placebo (*p* < .001) and control (*p* < .001) primes. As expected, there was no significant difference following the placebo prime compared to the control (*p* = .518). There was also a significant main effect of time (*F* (1, 34) = 27.94, *p* < .001, *η*_*p*_^2^ = .45). As expected, BACs were significantly higher at end of session compared to post-drink. Finally, there was also a significant condition × time interaction (*F* (2, 68) = 3.95, *p* = .038, *η*_*p*_^2^ = .10) with significantly higher BACs following the alcohol prime (0.27 ± 0.09) compared to the placebo-alcohol (0.00 ± 0.00) and control (0.00 ± 0.00) at post-drink (*p* < .001). Following the taste test, BACs were also significantly higher at the end of the session following the alcohol prime (0.32 ± 0.09) compared to the placebo (0.02 ± 0.03; *p* < .001) and control (0.02 ± 0.04; *p* < .001). There was no difference between the placebo and control drinks at post-drink or end of session (*p* = .518). Analyses for subjective intoxication and estimation of units can be found in the supplementary materials.

We also hypothesised that deficits in inhibitory subprocesses would predict unique variance in beer consumed during the bogus taste test and that the effect of alcohol intoxication on beer consumed would be partially mediated by the different components of control. However, the effect of alcohol priming on SSRTs was weak and deficits in inhibitory subprocesses did not predict unique variance in beer consumption; therefore, these analyses are included in supplementary materials.

### Discussion

The current studies aimed to investigate the effect of alcohol cue exposure and alcohol intoxication on proactive slowing, reactive control, signal detection and subsequent craving and ad libitum alcohol consumption. In study 1, there were no impairments of proactive slowing or signal detection following alcohol cue exposure (compared to neutral cue exposure), and contrary to hypotheses, reactive control was unexpectedly faster following exposure to alcohol cues compared to neutral cues. Alcohol cues did have a weak effect on craving (on the obsessive scale of the AAAQ) and increased ad libitum alcohol consumption. In study 2, neither proactive slowing nor signal detection were impaired by alcohol intoxication. SSRTs were slower (indicative of worse inhibitory control) following alcohol compared to the placebo prime supporting our hypothesis, but there was no difference compared to the control condition. SSRTs were also significantly faster following the placebo compared to the control suggesting the anticipated effects of alcohol did not impair reactive control. As expected, alcohol priming did increase self-reported craving and ad libitum alcohol consumption (compared to placebo and control).

Taken together, these findings provide limited support for theoretical models which suggest that inhibitory control is a state variable which fluctuates in response to internal (alcohol intoxication) and environmental (cue exposure) events (de Wit 2009; Jones et al. 2013). Specifically, we failed to replicate numerous studies which have demonstrated impairments following alcohol cue exposure in both non-dependent (Field and Jones 2017; Kreusch et al. 2013; Monk et al. 2016; Petit et al. 2012; Weafer and Fillmore 2012) and dependent drinkers (Gauggel et al. 2010; Muraven and Shmueli 2006). Indeed, SSRTs were faster during alcohol cue exposure compared to neutral cue exposure when analysing both central and peripheral stop-signal blocks and there was no difference across central blocks only. However, a recent meta-analysis (Jones et al. 2018) demonstrated this effect is likely to be small in magnitude (standardised mean difference = 0.21^3^), and other research has also failed to demonstrate these effects across non-dependent and dependent drinkers (Field and Jones 2017; Nederkoorn et al. 2009; Weafer and Fillmore 2012).

Importantly, we demonstrated support that acute alcohol intoxication impaired reactive control compared to a placebo which supports previous research (e.g. Fillmore et al. 2009; Marczinski et al. 2005; Weafer and Fillmore 2008). However, the addition of a control group revealed that the effect of alcohol intoxication on SSRTs is limited. We also failed to support the observation that placebo intoxication impairs inhibitory control compared to control groups (Christiansen et al. 2016) as when analysing both central and peripheral blocks, SSRTs were unexpectedly faster following the placebo compared to the control, although there was no difference across central blocks only. These results may be partially explained by compensatory effects in which participants in the placebo condition may attempt to compensate for impairments (Fillmore et al. 1994), and research demonstrates that individuals who show larger compensatory effects following a placebo usually show more tolerance to impairment following alcohol (Testa et al. 2006). Furthermore, although Campbell et al. (2017) reported an impairment of motor (but not saccadic) inhibition following alcohol intoxication, their effect was smaller than predicted. This led them to suggest that there is a lack of power and the existence of publication bias in the literature. Similarly, Jones et al. (2018) also recently questioned the clinical significance of any impairments due to the small effect size and lack of associations with substance use behaviours.

Our findings provide support for recent cognitive models which suggest that inhibitory control is a multi-process behaviour (Verbruggen et al. 2014a). We were able to adapt tasks from the literature to isolate signal detection and proactive control, and across both studies showed that heavy drinkers demonstrate proactive slowing when inhibition is more likely and also increased stopping times when stop signals are in the periphery, which demonstrates the contribution of signal detection to reactive stopping processes. Notably, the requirement of participants to detect a visual central or peripheral stop signal and differentiate between natural and man-made words may have improved the ecological validity of the task as in the real world, signal detection and response inhibition occur under complex conditions (e.g., multiple environmental demands) and in ‘noisy’ surroundings (Verbruggen et al. 2014b). However, this may have contributed to a failure to replicate previous findings due to the increased task difficulty and, therefore, attention requirements. The use of a visual stop signal did, however, decrease the need for divided attention as this was the same modality as the go stimuli (Verbruggen et al. 2014b). Furthermore, it should be noted that Campbell et al. (2017) also failed to demonstrate a reliable decrease in proactive slowing following alcohol priming; however, as previously noted, there is a lack of research focusing on this aspect of executive control and therefore it is still possible that proactive slowing is impaired by alcohol. Despite limited evidence for impairments *within* individuals, future research should therefore investigate whether these impairments are exacerbated in clinical populations, or evident in individuals who do not drink to hazardous levels (Sharma 2017).

Finally, our findings provide further empirical support of studies which have demonstrated that alcohol-related cues (Fatseas et al. 2015; Koordeman et al. 2011; MacKillop and Lisman 2007) and alcohol intoxication (e.g. Christiansen et al. 2012; De Wit and Chutuape 1993; Rose and Grunsell 2008) increase subsequent alcohol seeking. Furthermore, although the placebo-alcohol increased subjective feelings of lightheadedness supporting previous research (e.g. Rose et al. 2013), there was no difference in beer consumption following the placebo-alcohol and control as predicted. Nevertheless, this replicates the findings of Christiansen et al. (2012) and implies that the pharmacological effects (not the anticipated effects) of alcohol are key to the priming effect on subsequent motivation to consume alcohol. However, those studies (e.g. Marlatt et al. 1973) which have found an increase in alcohol consumption following a placebo compared to a control tend to have a short interval between administration of the drinks and the taste test. In both Christiansen et al. (2012) and the current study, there was a longer interval (approximately 40 min passed between beverage consumption, the stop-signal task, and the bogus taste test in the current study); therefore, the effect of the placebo on subsequent motivation to drink may have reduced over time (Christiansen et al. 2012). Additionally, despite the increase in ad libitum consumption in both studies, we did not demonstrate robust increases in craving. Although contradictory to our hypothesis and previous findings (e.g. Christiansen et al. 2012; Fatseas et al. 2015; Field and Jones 2017; Rose et al. 2013), this suggests that alcohol seeking can increase without an accompanied increase in self-reported craving, which has also been reported in previous studies (e.g. Wiers et al. 2010; see also Tiffany 1990; Wiers et al. 2007).

Our findings should be interpreted in light of limitations. In study 1, our cue exposure manipulation may not have been strong enough to influence inhibitory control. Although we used similar methods to Field and Jones (2017), their manipulation may have been strengthened by asking participants to sniff beer after every 16 trials rather than at the beginning of each block, and responding directly to alcohol-related cues (rather than neutral words). Additionally, their sample had greater levels of weekly alcohol consumption (~ 34.18 units) and AUDIT scores (~ 14.18), suggesting these individuals demonstrate a greater sensitivity to cue reactivity (Herrmann et al. 2001). Second, we are unable to separate the effects of these different cue modalities on inhibitory processes and ad libitum alcohol consumption and future studies should attempt to isolate these effects (Monk et al. 2016).

In conclusion, alcohol-related cues and alcohol priming increase motivation to consume subsequent alcohol; however, this is unlikely due to an impairment in the ability to inhibit behaviour(s). Future research should attempt to clarify the mechanisms underlying this relationship and investigate additional processes which may lead to impairments in inhibitory control, in order to increase our understanding of hazardous drinking.

## Electronic supplementary material

ESM 1 (DOCX 25688 kb)

## Footnotes

1 Two participants were identified during the outlier analysis with a high frequency of errors. However, their removal did not change the pattern of results.

2 We also predicted that the placebo-alcohol beverage would increase subjective intoxication ratings, motivation to drink, beer consumed in the taste test and deficits in proactive and reactive control compared to the control condition, but not to the same extent as alcohol.

3 Note that this meta-analysis was published prior to recruitment of this study, hence the larger estimate of *d* = .39 used for the power calculation.

## References

[CR1] Aron AR (2011). From reactive to proactive and selective control: developing a richer model for stopping inappropriate responses. Biol Psychiatry.

[CR2] Brevers D, Bechara A, Kilts C, Antoniali V, Bruylant A, Verbanck P, Kornreich C, Noël X (2017). Competing motivations: proactive response inhibition toward addiction-related stimuli in quitting-motivated individuals. J Gambl Stud.

[CR3] Campbell AE, Chambers CD, Allen CPG, Hedge C, Sumner P (2017). Impairment of manual but not saccadic response inhibition following acute alcohol intoxication. Drug Alcohol Depend.

[CR4] Christiansen P, Rose AK, Cole JC, Field M (2012). A comparison of the anticipated and pharmacological effects of alcohol on cognitive bias, executive function, craving and ad-lib drinking. J Psychopharmacol.

[CR5] Christiansen P, Jennings E, Rose AK (2016). Anticipated effects of alcohol stimulate craving and impair inhibitory control. Psychol Addict Behav.

[CR6] Colder CR, O'Connor R (2002). Attention bias and disinhibited behavior as predictors of alcohol use and enhancement reasons for drinking. Psychol Addict Behav.

[CR7] Collins RL, Lapp WM (1992). The temptation and restraint inventory for measuring drinking restraint. Br J Addict.

[CR8] Czapla M, Simon JJ, Friederich HC, Herpertz SC, Zimmermann P, Loeber S (2015). Is binge drinking in young adults associated with an alcohol-specific impairment of response inhibition?. Eur Addict Res.

[CR9] de Wit H (2009). Impulsivity as a determinant and consequence of drug use: a review of underlying processes. Addict Biol.

[CR10] De Wit H, Chutuape MA (1993). Increased ethanol choice in social drinkers following ethanol preload. Behav Pharmacol.

[CR11] Duka T, Tasker R, Stephens DN (1998). Alcohol choice and outcome expectancies in social drinkers. Behav Pharmacol.

[CR12] Elchlepp H, Lavric A, Chambers CD, Verbruggen F (2016). Proactive inhibitory control: a general biasing account. Cogn Psychol.

[CR13] Fatseas M, Serre F, Alexandre JM, Debrabant R, Auriacombe M, Swendsen J (2015). Craving and substance use among patients with alcohol, tobacco, cannabis or heroin addiction: a comparison of substance- and person-specific cues. Addiction.

[CR14] Field M, Jones A (2017). Elevated alcohol consumption following alcohol cue exposure is partially mediated by reduced inhibitory control and increased craving. Psychopharmacology.

[CR15] Fillmore MT (2003). Drug abuse as a problem of impaired control: current approaches and findings. Behav Cogn Neurosci Rev.

[CR16] Fillmore M, Mulvihill LE, Vogel-Sprott M (1994). The expected drug and its expected effect interact to determine placebo responses to alcohol and caffeine. Psychopharmacology.

[CR17] Fillmore M, Ostling EW, Martin CA, Kelly TH (2009). Acute effects of alcohol on inhibitory control and information processing in high and low sensation-seekers. Drug Alcohol Depend.

[CR18] Gauggel S, Heusinger A, Forkmann T, Boecker M, Lindenmeyer J, Miles Cox W, Staedtgen M (2010). Effects of alcohol cue exposure on response inhibition in detoxified alcohol-dependent patients. Alcohol Clin Exp Res.

[CR19] Goldstein RZ, Volkow ND (2002). Drug addiction and its underlying neurobiological basis: neuroimaging evidence for the involvement of the frontal cortex. Am J Psychiatry.

[CR20] Hagger MS, Chatzisarantis NLD, Alberts H, Anggono CO, Batailler C, Birt AR, Brand R (2016). A multilab preregistered replication of the ego-depletion effect. Perspect Psychol Sci.

[CR21] Ham LS, Stewart SH, Norton PJ, Hope DA (2005). Psychometric assessment of the comprehensive effects of alcohol questionnaire: comparing a brief version to the original full scale. J Psychopathol Behav Assess.

[CR22] Herrmann MJ, Weijers HG, Wiesbeck GA, Böning J, Fallgatter AJ (2001). Alcohol cue-reactivity in heavy and light social drinkers as revealed by event-related potentials. Alcohol Alcohol.

[CR23] Hofmann W, Schmeichel BJ, Baddeley AD (2012). Executive functions and self-regulation. Trends Cogn Sci.

[CR24] Houston RJ, Derrick JL, Leonard KE, Testa M, Quigley BM, Kubiak A (2014). Effects of heavy drinking on executive cognitive functioning in a community sample. Addict Behav.

[CR25] Jones A, Christiansen P, Nederkoorn C, Houben K, Field M (2013). Fluctuating disinhibition: implications for the understanding and treatment of alcohol and other substance use disorders. Front Psych.

[CR26] Jones A, Robinson E, Duckworth J, Kersbergen I, Clarke N, Field M (2018). The effects of exposure to appetitive cues on inhibitory control: a meta-analytic investigation. Appetite.

[CR27] Kamarajan C, Porjesz B, Jones KA, Choi K, Chorlian DB, Padmanabhapillai A, Rangaswamy M, Stimus AT, Begleiter H (2005). Alcoholism is a disinhibitory disorder: neurophysiological evidence from a go/no-go task. Biol Psychol.

[CR28] Koordeman R, Anschutz DJ, Engels RCME (2011). Exposure to alcohol commercials in movie theaters affects actual alcohol consumption in young adult high weekly drinkers: an experimental study. Am J Addict.

[CR29] Kreusch F, Vilenne A, Quartemont E (2013). Response inhibition toward alcohol-related cues using an alcohol go/no-go task in problem and non-problem drinkers. Addict Behav.

[CR30] Lejuez CW, Aklin WM, Zvolensky MJ, Pedulla CM (2003). Evaluation of the Balloon Analogue Risk Task (BART) as a predictor of adolescent real-world risk-taking behaviours. J Adolesc.

[CR31] Logan GD, Cowan WB, Davis KA (1984). On the ability to inhibit simple and choice reaction time responses: a model and a method. J Exp Psychol Hum Percept Perform.

[CR32] MacKillop J, Lisman SA (2007). Examining the effect of perceived availability on craving for alcohol: a quasi-experimental approach. Addict Res Theory.

[CR33] Marczinski CA, Abroms BD, Van Selst M, Fillmore MT (2005). Alcohol-induced impairment of behavioral control: differential effects on engaging vs. disengaging responses. Psychopharmacology.

[CR34] Marlatt GA, Demming B, Reid JB (1973). Loss of control drinking in alcoholics: an experimental analogue. J Abnorm Psychol.

[CR35] McEvoy PM, Stritzke WGK, French DJ, Lang AR, Ketterman RL (2004). Comparison of three models of alcohol craving in young adults: a cross-validation. Addiction.

[CR36] Monk RL, Sunley J, Qureshi AW, Heim D (2016). Smells like inhibition: the effects of olfactory and visual alcohol cues on inhibitory control. Psychopharmacology.

[CR37] Muraven M, Shmueli D (2006). The self-control costs of fighting the temptation to drink. Psychol Addict Behav.

[CR38] Nederkoorn C, Baltus M, Guerrieri R, Wiers RW (2009). Heavy drinking is associated with deficient response inhibition in women but not in men. Pharmacol Biochem Behav.

[CR39] Nigg JT, Wong MM, Martel MM, Jester JM, Puttler LI, Glass JM, Adams KM, Fitzgerald HE, Zucker RA (2006). Poor response inhibition as a predictor of problem drinking and illicit drug use in adolescents at risk for alcoholism and other substance use disorders. J Am Acad Child Adolesc Psychiatry.

[CR40] Patton JH, Stanford MS, Barratt ES (1995). Factor structure of the Barratt Impulsiveness Scale. J Clin Psychol.

[CR41] Petit G, Kornreich C, Noel X, Verbanck P, Campanella S (2012). Alcohol-related context modulates performance of social drinkers in a visual go/no-go task: a preliminary assessment of event-related potentials. PLoS One.

[CR42] Plawecki MH, Koskie S, Kosobud A, Justiss MD, O'Connor S (2018). Alcohol intoxication progressively impairs drivers’ capacity to detect important environmental stimuli. Pharmacol Biochem Behav.

[CR43] Roberts W, Miller MA, Weafer J, Fillmore MT (2014). Heavy drinking and the role of inhibitory control of attention. Exp Clin Psychopharmacol.

[CR44] Rose AK, Duka T (2007). The influence of alcohol on basic motoric and cognitive disinhibition. Alcohol Alcohol.

[CR45] Rose AK, Grunsell L (2008). The subjective, rather than the disinhibiting, effects of alcohol are related to binge drinking. Alcohol Clin Exp Res.

[CR46] Rose AK, Hobbs M, Drummond C (2013). Differentiating the contribution of pharmacological from alcohol expectancy effects to changes in subjective response and priming over successive drinks. Alcohol Clin Exp Res.

[CR47] Rubio G, Jimenez M, Rodriguez-Jimenez R, Martinez I, Avila C, Ferre F, Jimenez-Arriero MA, Ponce G, Palomo T (2008). The role of behavioral impulsivity in the development of alcohol dependence: a 4-year follow-up study. Alcohol Clin Exp Res.

[CR48] Rupp CI, Beck JK, Heinz A, Kemmler G, Manz S, Tempel K, Fleischhacker WW (2016). Impulsivity and alcohol dependence treatment completion: is there a neurocognitive risk factor at treatment entry?. Alcohol Clin Exp Res.

[CR49] Saunders JB, Aasland OG, Babor TF, De la Fuente JR, Grant M (1993). Development of the alcohol use disorders identification test (AUDIT): WHO collaborative project on early detection of persons with harmful alcohol consumption II. Addiction.

[CR50] Scholz U, La Marca R, Nater UM, Aberle I, Ehlert U, Hornung R, Martin M, Kliegel M (2009). Go no-go performance under psychosocial stress: beneficial effects of implementation intentions. Neurobiol Learn Mem.

[CR51] Sharma D (2017). The variable nature of cognitive control in a university sample of young adult drinkers. J Appl Soc Psychol.

[CR52] Smith J, Mattick R, Jamadar S, Iredale J (2014). Deficits in behavioural inhibition in substance abuse and addiction: a meta-analysis. Drug Alcohol Depend.

[CR53] Sobell LC, Sobell MB (1990). Self-report issues in alcohol abuse: state of the art and future directions. Behav Assess.

[CR54] Testa M, Fillmore MT, Norris J, Abbey A, Curtin JJ, Leonard KE, Mariano KA, Thomas MC, Nomensen KJ, George WH, Vanzile-Tamsen C, Livingston JA, Saenz C, Buck PO, Zawacki T, Parkhill MR, Jacques AJ, Hayman LW (2006). Understanding alcohol expectancy effects: revisiting the placebo condition. Alcohol Clin Exp Res.

[CR55] Tiffany ST (1990). A cognitive model of drug urges and drug-use behavior: role of automatic and nonautomatic processes. Psychol Rev.

[CR56] Townshend JM, Duka T (2001). Attentional bias associated with alcohol cues: differences between heavy and occasional social drinkers. Psychopharmacology.

[CR57] Verbruggen F, De Houwer J (2007). Do emotional stimuli interfere with response inhibition? Evidence from the stop signal paradigm. Cognit Emot.

[CR58] Verbruggen F, Logan GD (2009). Models of response inhibition in the stop-signal and stop-change paradigms. Neurosci Biobehav Rev.

[CR59] Verbruggen F, Logan GD (2009). Proactive adjustments of response strategies in the stop-signal paradigm. J Exp Psychol Hum Percept Perform.

[CR60] Verbruggen F, Chambers CD, Logan GD (2013). Fictitious inhibitory differences: how skewness and slowing distort the estimation of stopping latencies. Psychol Sci.

[CR61] Verbruggen F, McLaren IPL, Chambers CD (2014). Banishing the control homunculi in studies of action control and behavior change. Perspect Psychol Sci.

[CR62] Verbruggen F, Stevens T, Chambers CD (2014). Proactive and reactive stopping when distracted: an attentional account. J Exp Psychol Hum Percept Perform.

[CR63] Weafer J, Fillmore MT (2008). Individual differences in acute alcohol impairment of inhibitory control predict ad libitum alcohol consumption. Psychopharmacology.

[CR64] Weafer J, Fillmore MT (2012). Alcohol-related stimuli reduce inhibitory control of behavior in drinkers. Psychopharmacology.

[CR65] Weafer J, Fillmore MT (2016). Low-dose alcohol effects on measures of inhibitory control, delay discounting, and risk-taking. Curr Addict Rep.

[CR66] Wiers RW, Bartholow BD, van den Wildenberg E, Thush C, Engels RC, Sher KJ, Grenard J, Ames SL, Stacy AW (2007). Automatic and controlled processes and the development of addictive behaviors in adolescents: a review and a model. Pharmacol Biochem Behav.

[CR67] Wiers RW, Rinck M, Kordts R, Houben K, Strack F (2010). Retraining automatic action-tendencies to approach alcohol in hazardous drinkers. Addiction.

[CR68] Yücel M, Oldenhof E, Ahmed S, Belin D, Billieux J, Bowden‐Jones H, Carter A et al (2018) A transdiagnostic dimensional approach towards a neuropsychological assessment for addiction: an international Delphi consensus study. Addiction. 10.1111/add.14424 10.1111/add.14424 PMC6386631 30133930

[CR69] Zandbelt BB, Van Buuren M, Kahn RS, Vink M (2011). Reduced proactive inhibition in schizophrenia is related to corticostriatal dysfunction and poor working memory. Biol Psychiatry.

