# Supplementary material for: The effect of alcohol cue exposure and acute intoxication on inhibitory control processes and ad libitum alcohol consumption
Source: Psychopharmacology (Berl). 2019 Mar 27;236(7):2187–99. doi: 10.1007/s00213-019-05212-4 (PMC6647270; doi:10.1007/s00213-019-05212-4)
Supplement: Supplementary file 1 — (DOCX 25688 kb) [file 213_2019_5212_MOESM1_ESM.docx]

**Supplementary Information**

**The effect of alcohol cue-exposure and acute intoxication on inhibitory control processes and *ad libitum* alcohol consumption.**

Laura Baines ^1 2^ 🖃 Matt Field ^2 3^ Paul Christiansen ^1 2^ Andrew Jones ^1 2^

**Methods**

*Balloon Analogue Risk Task (BART; ((Lejuez et al. 2003))*

In both studies, participants completed a short cognitive task in which they had to click a mouse to pump up simulated balloons. They were presented with one balloon per trial and completed 10 trials. Each time participants clicked to pump up the balloon, the balloon increased in size and they hypothetically collected $0.05 in a temporary bank. They could transfer this money to a “permanent” bank by clicking collect. However, they were informed that if the balloon bursts, they would lose the money stored in the temporary bank. Once the balloon had burst or the participant had collected the money, a new trial began whereby the size of the balloon was reset and the temporary bank was set back to $0. We programmed the balloons to burst on a variable ratio, with 64 pumps as the average explosion point. Participants completed this task after the bogus taste test in both studies; they were told that alcohol would impair their performance on this task in which they had the opportunity to win small amounts of money in order to increase their motivation to reduce their intake (see (Christiansen et al 2012; Field & Jones 2017)). Actual performance on this task was of secondary importance but data is available upon request.

**Results**

*Sample characteristics (see table 1)*

We conducted independent samples t-tests to compare the participants in study 1 (alcohol-cue exposure) to participants in study 2 (alcohol priming) on baseline variables and drinking variables. There were no significant differences in the age of participants (*t* (98) = -.56, p= .575, d= .12), AUDIT scores (*t* (98) = .83, p= .408, d= .17), units consumed in the two weeks prior to the study (*t* (98) = .70, p= .485, d= .15) or total scores on the Barratt impulsivity scale (*t* (98) = .27, p= .788, d= .06).

*Table 1: Sample characteristics and baseline variables of participant samples in study 1 and study 2 split by gender (values are mean, SD)*

Study 1 Study 2

Males Females Sample Males Females Sample

N 27 37 64 19 17 36

Age 22.04(9.26) 24.97(9.30) 23.73(9.33) 26.11(8.05) 23.24(6.33) 24.75(7.33)

AUDIT 13.48(5.21) 11.95(4.16) 12.59(4.65) 11.32(3.89) 12.29(5.75) 11.78(4.81)

TLFB 68.87(46.16) 42.53(19.56) 53.64(35.64) 60.32(25.68) 36.15(19.43) 48.90(25.72)

BIS 69.81(10.54) 65.22(8.40) 67.16(9.56) 66.00(11.68) 69.65(9.97) 67.72(10.91)

*Audit=Alcohol use disorder identification test. Scores above 8 are indicative of hazardous drinking. TLFB=timeline follow back. Units consumed in 14 days prior to taking part. BIS=Total scores on Barratt impulsivity scale.*

***Study 1***

*Hypothesis 2: Does alcohol-cue exposure increase craving and ad-libitum alcohol consumption?*

Participants also had marginally significantly higher BACs following alcohol cue-exposure (0.03 ± 0.04) compared to neutral cue-exposure (0.02 ± 0.03; *t* (62) = 2.00, p=.05, *d*= 0.33).

*Hypothesis 3: Do deficits in proactive slowing and signal detection predict unique variance in alcohol consumption after controlling for reactive inhibition?*

We conducted multiple regression analyses on each condition separately. The full regression model did not predict a significant amount of variance (R² = .05) in beer consumed (as a percentage of total fluid consumed) following alcohol-cue exposure (F (3, 57) = 0.90, p= .447). SSRT (β= .22, p= .154), signal detection (β= -.07, p= .593) or proactive slowing (β= -.01, p= .958) were not significant predictors. Similarly, the overall regression model did not predict a significant level of variance (R² =.12) in beer consumed following neutral cue-exposure (F (3, 57) = 2.29, p= .088). Again, neither SSRT (β= -.18, p= .219), signal detection (β= -.19, p= .196) or proactive slowing (β= -.09, p= .498) were significant predictors.

*Hypothesis 5: The effects of alcohol cues on ad libitum alcohol consumption will be partially mediated by changes in the different components of control (see figure 1-3).*

To examine whether changes in the different components of control partially mediate the effect of alcohol-cue exposure on *ad libitum* alcohol consumption, we ran a within-subjects mediation analysis using MEMORE macro for SPSS (Montoya and Hayes 2016). We used bias-corrected, bootstrapped (1000 samples) confidence intervals. Firstly, there was no indirect effect of alcohol-cue exposure on beer consumed during the bogus taste test via SSRT (B= -1.02 (SE= 1.00), 95% CI -3.47 to 0.63). However, the direct effect of alcohol-cue exposure on beer consumed was significant after controlling for SSRT (B= 7.80 (SE= 2.50), 95% CI 2.80 to 12.79). Secondly, there was also no indirect effect of alcohol-cue exposure on *ad libitum* consumption via proactive slowing (B= 0.46 (SE= 0.69), 95% CI -0.30 to 2.79), although there was a direct effect after controlling for proactive slowing (B=6.32 (SE= 2.48), 95% CI 1.36 to 11.28). Thirdly, there was no indirect effect via signal detection (B= 0.31 (SE= 0.79), 95% CI -0.76 to 2.78), however the direct effect was significant after controlling for signal detection (B= 6.46 (SE= 2.50), 95% CI 1.45 to 11.47). Lastly, there was a significant total effect of alcohol cue-exposure on *ad libitum* beer consumption (B= 6.77 (SE= 2.46), 95% CI 1.86 to 11.69).

**Fig 1: The direct and indirect effect of alcohol-cue exposure on ad libitum alcohol consumption via SSRT.**

***
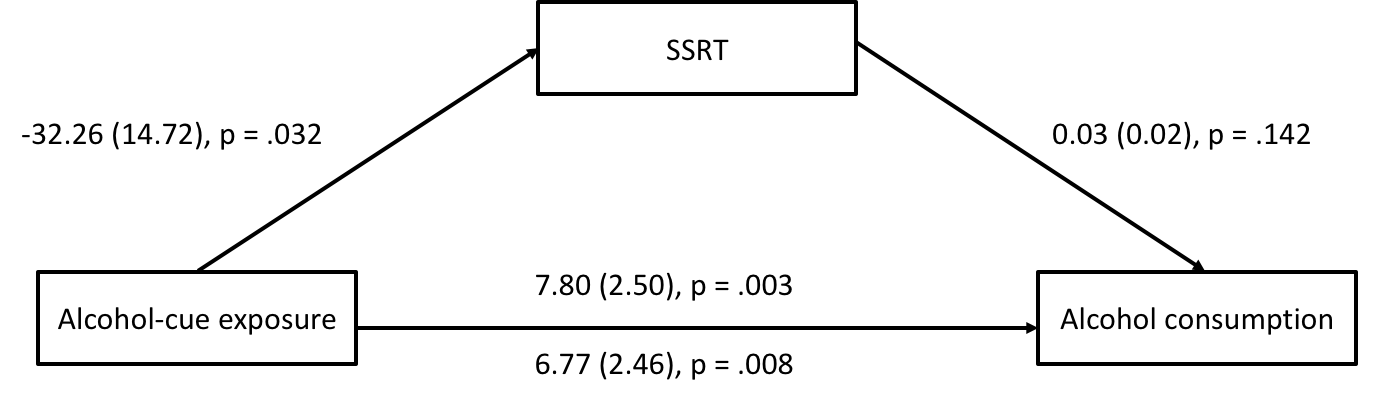
***

**Fig 2: The direct and indirect effect of alcohol-cue exposure on ad libitum alcohol consumption via proactive slowing.**


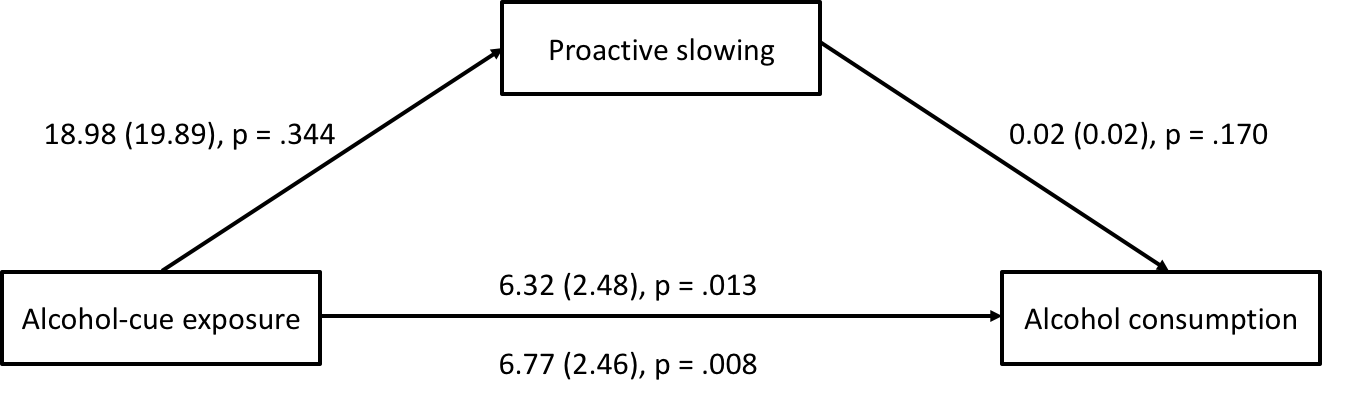


**Fig 3: The direct and indirect effect of alcohol-cue exposure on ad libitum alcohol consumption via signal detection.**

***
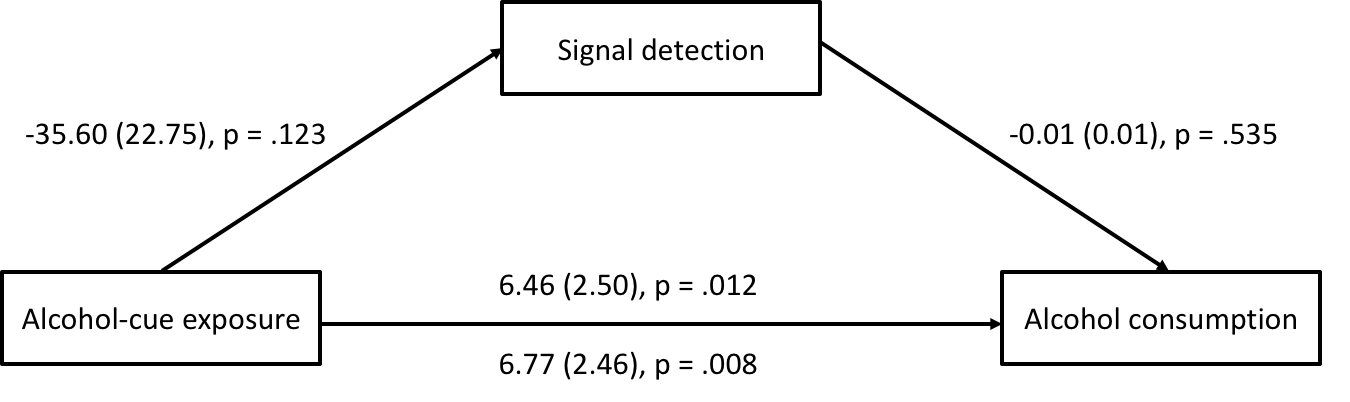
***

***Study 2***

*Hypothesis 2: Does alcohol intoxication increase alcohol-seeking measures*

There was a significant main effect of condition of subjective feelings of light-headedness (F (2, 70) = 39.23, p < .001, η_p_^2^= .53). Participants felt significantly more light headed following the alcohol priming drink (38.97 ±27.82) compared to the control (2.40 ±5.49; p < .001) and placebo-alcohol drink (12.89 ±19.69; p < .001). Participants also reported feeling significantly more light headed following the placebo-alcohol drink compared to the control (p= .004). There was also a significant main effect of condition of subjective feelings of alertness, (F (2, 60) = 10.61, p < .001, η_p_^2^= .23). Participants reported feeling significantly more alert following the control drink (65.71, ±26.10) compared to the alcohol (42.44 ±26.17; p< .001) and placebo-alcohol drinks (54.56 ±23.10; p = .023). Participants also reported feeling significantly more alert following the placebo-alcohol drink compared to the alcohol drink (p = .008). There were no other significant differences in the subjective intoxication measures between sessions (ps >.05).

There was a significant main effect of condition on estimation of units in the priming drink, (F (2, 60) = 92.84, p < .001, η_p_^2^= .73). Participants thought they had consumed significantly more units in the alcohol drink (4.61 ±2.38) compared to the placebo-alcohol (2.69 ±1.63; p < .001) and control drink (0.00 ±0.00; p < .001). They also reported consuming significantly more units in the placebo-alcohol drink compared to the control drink (p < .001). Notably, two participants believed that the placebo drink contained no alcohol but removal of these did not significantly influence our results.

*Hypothesis 4: Do inhibitory sub processes predict variance in beer consumed during bogus taste test.*

We ran multiple regression analyses on each condition separately. The full regression model did not predict a significant amount of variance in beer consumed (as a percentage of total fluid consumed) in the alcohol session (R^2^ = 0.06, F (3, 29) = 0.64, p= .598). SSRT (β= -.33, p= .195), signal detection (β= .25, p= .323) and proactive slowing (β= .10, p= .608) were not significant predictors of beer consumed following alcohol intoxication. The full regression model also did not predict a significant amount of variance in beer consumed during the placebo session (R^2^ = .08, F (3, 29) = 0.89, p= .457). Neither SSRT (β = .20, p = .339), signal detection (β =-.26, p = .186) nor proactive slowing (β = .03, p= .90) were significant predictors of beer consumed following the placebo-alcohol prime. Lastly, the full regression model also did not predict significant variance in beer consumed during the control session (R^2^ = .01, F (3, 29) = 0.06, p= .982). Again, neither SSRT (β = .06, p= .839), signal detection (β = -.10, p= .729) nor proactive slowing (β = .02, p =.922) were significant predictors of beer consumed following the control prime.

*Hypothesis 5: the effect of alcohol intoxication on beer consumed would be partially mediated by the different components of control.*

*Alcohol-control priming (see figure 4-6)*

There was no indirect effect of alcohol priming (compared to the control) on *ad libitum* consumption via SSRT (B = -.20 (SE = 0.99), 95% CI -5.58 to 0.79). However, the direct effect of alcohol priming on consumption was significant after controlling for SSRT (B = 13.02 (SE = 4.04), 95% CI 4.76 to 21.28). Similarly, there was no indirect effect of alcohol priming on consumption via proactive slowing (B = 0.77 (SE = 1.15), 95% CI -0.67 to 4.06), although the direct effect was significant after controlling for proactive slowing (B= 12.05 (SE = 4.05), 95% CI 3.77 to 20.32). Thirdly, there was no indirect effect via signal detection (B = 0.15 (SE= 1.14), 95% CI -1.73 to 3.07), however, the direct effect was significant after controlling for signal detection (B= 12.67 (SE = 4.10), 95% CI 4.30 to 21.03). Lastly, there was a significant total effect (B = 12.82 (SE = 3.94), 95% CI 4.80 to 20.84) of alcohol priming on *ad libitum* alcohol consumption.

**Fig 4: The direct and indirect effect of alcohol priming (control-alcohol) on ad libitum alcohol consumption via SSRT.**


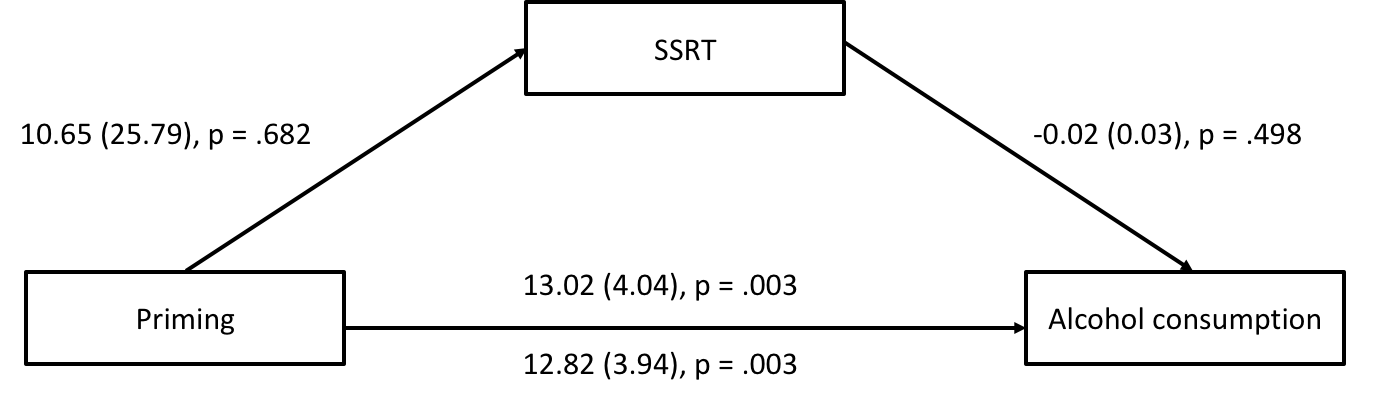


**Fig 5: The direct and indirect effect of alcohol priming (control-alcohol) on ad libitum alcohol consumption via Proactive slowing.**


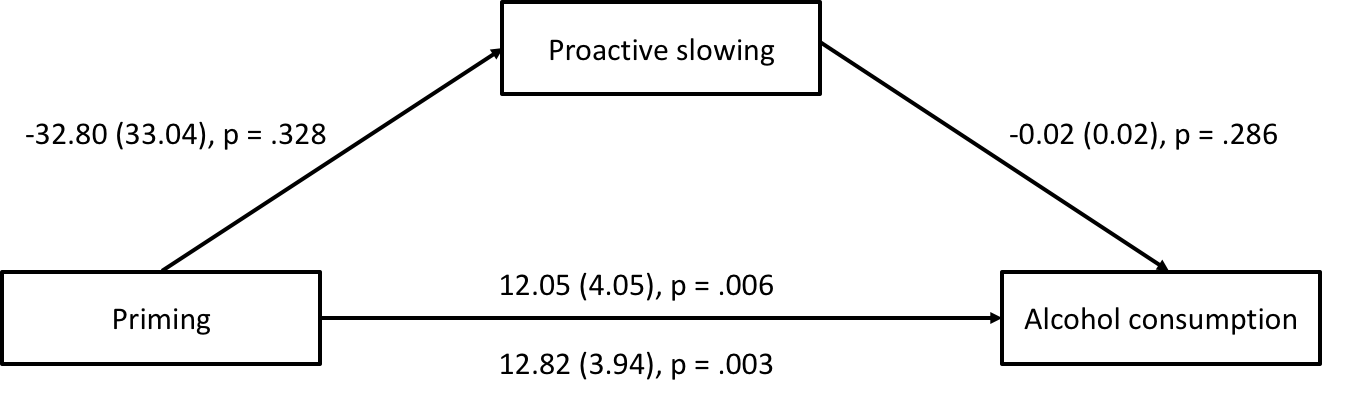


**Fig 6: The direct and indirect effect of alcohol priming (control-alcohol) on ad libitum alcohol consumption via Signal detection.**


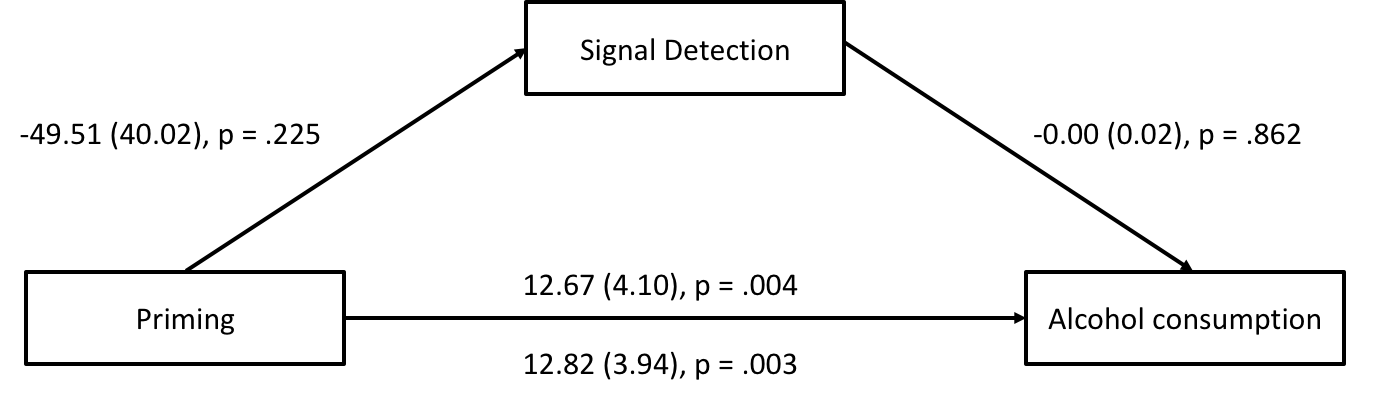


*Alcohol-placebo priming (see figure 7-9)*

There was no indirect effect of priming (alcohol compared to placebo) on beer consumed via SSRT (B = 1.27 (SE = 2.96), 95% CI -2.04 to 11.09) or direct effect of priming on beer consumed after controlling for SSRT (B= 6.84 (SE= 4.72), 95% CI -2.80 to 16.47). There was also no indirect effect of priming on beer consumed via proactive slowing (B = -0.04 (SE = 0.82), 95% CI -2.24 to 1.06) or no direct effect after controlling for proactive slowing (B = 8.14 (SE = 4.21), 95% CI -0.46 to 16.74). Additionally, there was no indirect effect of priming on beer consumed via signal detection (B= 0.01 (SE = 0.87), 95% CI -1.85 to 1.89) and no direct effect after controlling for signal detection (B= 8.09 (SE = 4.23), 95% CI -0.55 to 16.74). Lastly, there was no total effect of priming on beer consumed (B = 8.10 (SE = 4.09), 95% CI -0.23 to 16.44).

**Fig 7: The direct and indirect effect of priming (alcohol-placebo) on ad libitum alcohol consumption via SSRT.**


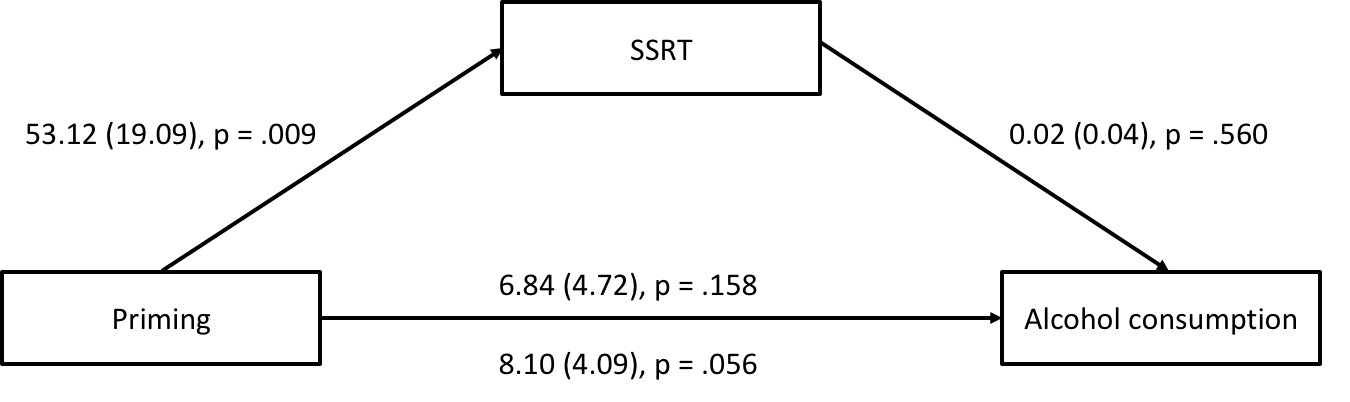


**Fig 8: The direct and indirect effect of priming (alcohol-placebo) on ad libitum alcohol consumption via proactive slowing.**


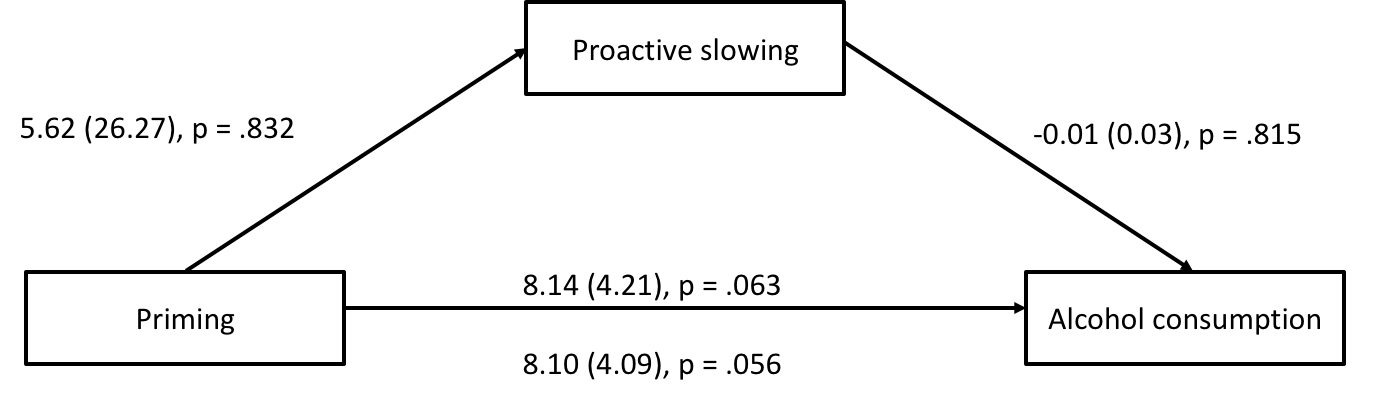


**Fig 9: The direct and indirect effect of priming (alcohol-placebo) on ad libitum alcohol consumption via signal detection.**

**
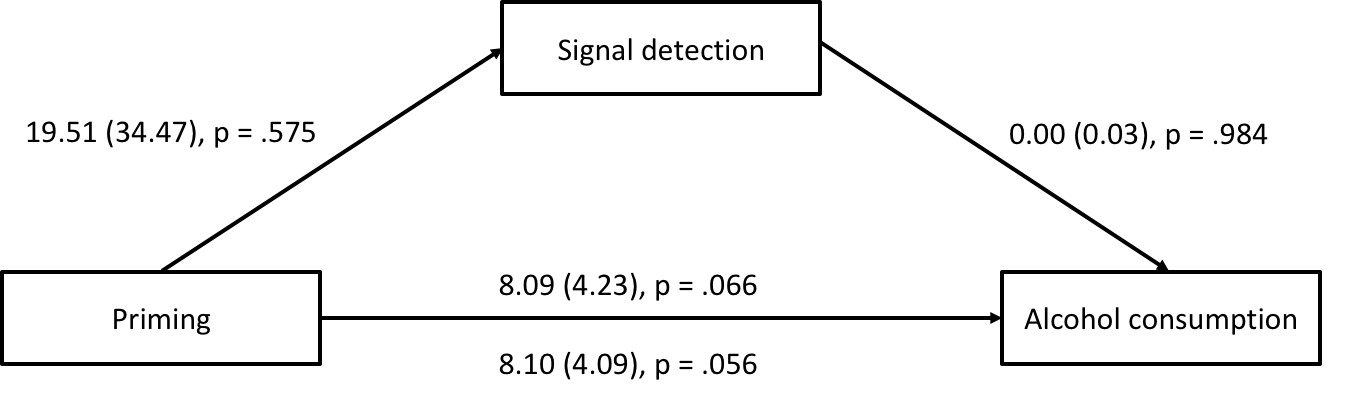
**

*Placebo-control priming (see figure 10-12).*

There was no indirect effect of priming (placebo compared to control) on consumption via SSRT (B= -0.44 (SE= 1.58), 95% CI -3.96 to 2.60) or no direct effect of priming on consumption after controlling for SSRT (B = 5.16 (SE = 4.00), 95% CI -3.01 to 13.33). There was also no indirect effect via proactive slowing (B = 0.12 (SE = 1.00), 95% CI -1.60 to 2.17) or direct effect after controlling for proactive slowing (B = 4.60 (SE = 3.87), 95% CI -3.31 to 12.50). Thirdly, there was no indirect effect via signal detection (B = 2.23 (SE = 2.26), 95% CI -0.90 to 8.00) or direct effect after controlling for signal detection (B = 2.49 (SE = 4.09), 95% CI -5.86 to 10.83). Lastly, there was no significant total effect of priming on alcohol consumption (B = 4.72 (SE = 3.67), 95% CI -2.76 to 12.19).

**Fig 10: The direct and indirect effect of priming (placebo-control) on ad libitum alcohol consumption via SSRT.**

**
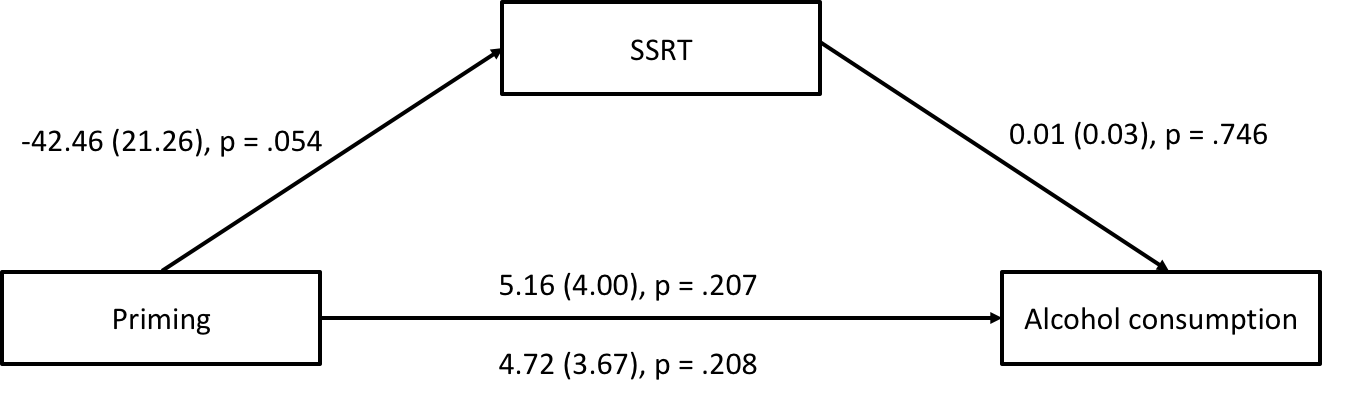
**

**Fig 11: The direct and indirect effect of priming (placebo-control) on ad libitum alcohol consumption via proactive slowing**.


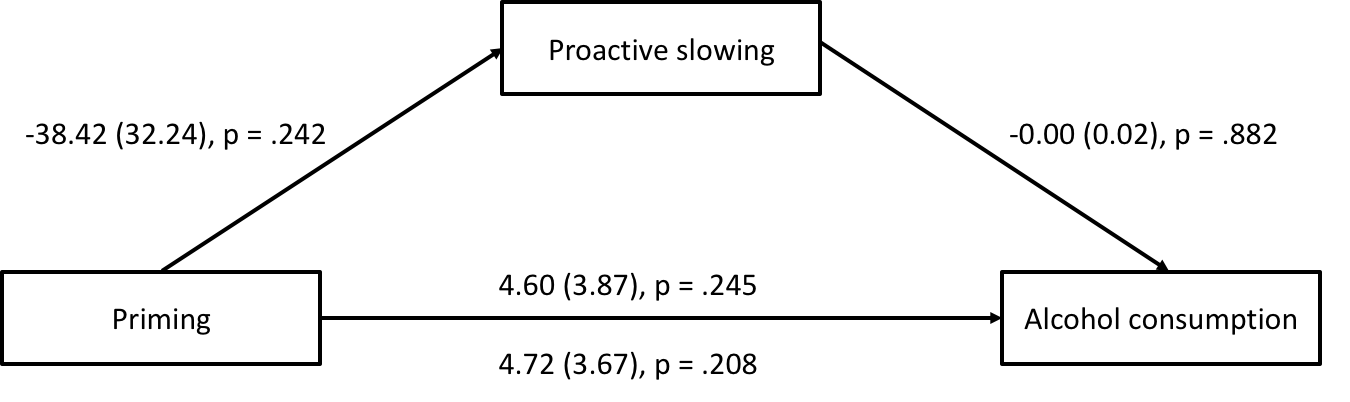


**Fig 12: The direct and indirect effect of priming (placebo-control) on ad libitum alcohol consumption via signal detection.**


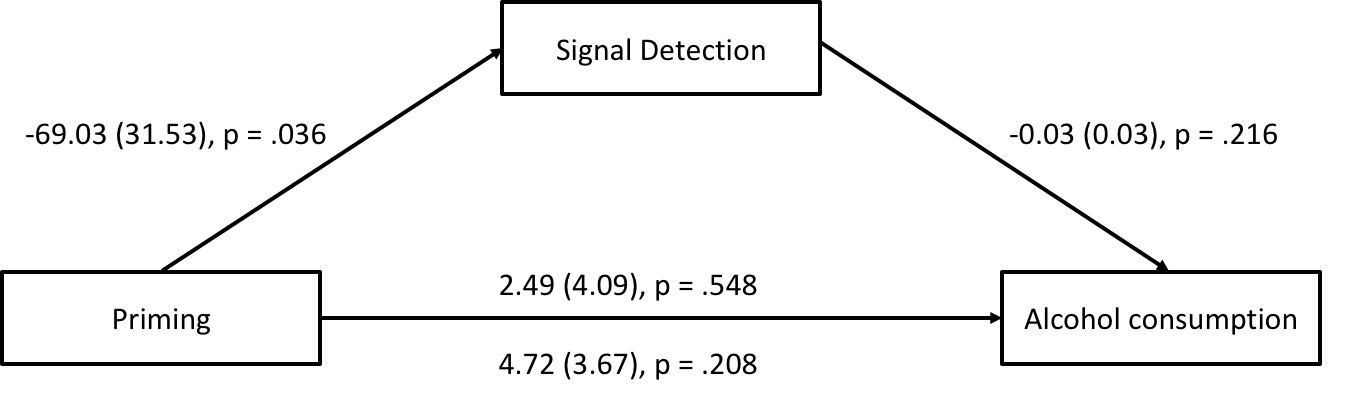


*Awareness of experimental aims*

In both studies we checked participants’ awareness of our experimental aims and none of the participants guessed the full aims (inferred from an open ended question). However, in study 1, 13 participants guessed the aim of the taste test was to measure how much they drank, but removing these had no significant effect on the results. Additionally, we removed 17 participants who correctly selected that the purpose of the computer task was to ‘*Assess my behavioural impulsivity (response inhibition).’* This removed the main effect of condition on SSRTs, however contrary to predictions this had shown SSRTs were faster following alcohol-cue exposure (compared to neutral-cue exposure). Similarly, in study 2, five participants guessed the aim of the taste test but when removed, the main effect of condition remained significant. Eight participants also correctly guessed the purpose of the computer task but removal of these only altered the main effect of condition on proactive slowing which had simply shown participants were slower to respond overall in the control priming session compared to the alcohol priming session.

*Exploratory analyses*

We also conducted exploratory analyses to investigate the effect of alcohol-cue exposure (study 1) and alcohol intoxication (study 2) on the number of errors made during the stop-signal task. This also allowed an investigation into differences in the number of errors between stop-signal blocks.

*Study 1*

To investigate the effect of alcohol-cue exposure on the number of errors made during the stop-signal task, we conducted a 2 (block: Central, Peripheral) x 2 (condition: Alcohol-cue exposure, Neutral-cue exposure) repeated measures ANOVA on incorrect no-signal trials and incorrect stop-signal trials. This showed no significant main effect of block on the number of incorrect no-signal responses (F (1, 61) = 0.15, p= .703, η_p_^2^= .002). There was however, a main effect of condition (F (1, 61) = 8.09, p= .006, η_p_^2^= .12) which revealed that participants made significantly less errors to no-signal trials in the bar laboratory compared to the neutral laboratory. Finally, there was no significant interaction between block and condition (F (1, 61) = 0.11, p= .746, η_p_^2^= .002). With regards to incorrect responses on stop-signal trials, there was a significant main effect of block (F (1, 61) = 10.72, p= .002, η_p_^2^= .15), which revealed that participants made more errors in the central blocks compared to the peripheral blocks. However, there was no main effect of condition (F (1, 61) = 0.84, p= .362, η_p_^2^= .01) or interaction (F (1, 61) = 2.21, p= .142, η_p_^2^= .04). Lastly, a paired samples t-test revealed no significant differences in incorrect responses in the no-signal block in the bar laboratory compared to the neutral laboratory (*t* (61) = -1.49, p= .141, d= .17).

*Table 2: The number of incorrect responses shown separately for each block of the SST and experimental condition (values are mean, SD).*

Alcohol cue-exposure Neutral cue-exposure

No-signal block 7.35 (4.97) 8.21 (4.83)

No-signal trials (central block) 6.06 (6.92) 7.74 (4.62)

Stop-signal trials (central block) 11.81 (4.40) 11.82 (3.66)

No-signal trials (periphery block) 5.68 (4.47) 7.69 (4.70)

Stop-signal trials (periphery block) 12.58 (4.53) 13.56 (5.24)

*Study 2*

We ran a 2 (block; Central, Peripheral) x 3 (condition: alcohol, placebo, control) repeated measures ANOVA on incorrect no-signal trials and incorrect stop-signal trials. This revealed there was no main effect of block on incorrect no-signal trials (F (1, 33) = 1.27, p= .269, η_p_^2^=.04), nor was there an effect of condition (F (2, 66) = 1.29, p= .284, η_p_^2^= .04) or an interaction (F (2, 66) = 0.01, p= .989, η_p_^2^= .00). With regards to incorrect stop-signal trials, there was a main effect of block (F (1, 33) = 9.39, p= .004, η_p_^2^= .22) which showed that participants made less errors in central blocks compared to periphery blocks. There was also a main effect of condition (F (2, 66) = 4.95, p= .01, η_p_^2^= .13) which demonstrated that participants made more errors on stop-signal trials following alcohol compared to the control (p= .014) and placebo-alcohol primes (p= .018). However, there was no significant difference following the control prime compared to the placebo-alcohol prime (p= .412). Furthermore, there was no significant interaction between block and condition (F (2, 66) = 1.28, p= .285, η_p_^2^= .04). Lastly, a repeated measures ANOVA revealed a main effect of condition on incorrect responses in the no-signal blocks (F (2, 54) = 14.24, p< .001, η_p_^2^= .30). However, this revealed that participants made significantly more errors during the control session compared to the alcohol (p< .001) and placebo (p< .001) sessions, but no difference in errors between the alcohol and placebo sessions (p= .525).

*Table 3: The number of incorrect responses shown separately for each block (no-signal, central stop-signal, peripheral stop-signal) of the SST and experimental condition (values are mean, SD).*

Control Alcohol Placebo

No-signal block 5.74 (3.66) 7.35 (5.31) 6.56 (6.24)

No-signal trials (central) 4.44 (2.94) 5.44 (5.05) 4.53 (4.61)

Stop-signal trials (central) 11.06 (2.91) 12.97 (3.52) 12.15 (2.54)

No-signal trials (periphery) 4.12 (3.01) 5.09 (4.51) 4.09 (3.54)

Stop-signal trials (periphery) 12.62 (4.53) 14.53 (5.23) 12.47 (3.74)

**References**

Christiansen P, Rose AK, Cole JC, Field M (2012) A comparison of the anticipated and pharmacological effects of alcohol on cognitive bias, executive function, craving and ad-lib drinking. Journal of Psychopharmacology 27: 84-92.

Field M, Jones A (2017) Elevated alcohol consumption following alcohol cue exposure is partially mediated by reduced inhibitory control and increased craving. Psychopharmacology (Berl) 234: 2979-2988.

Lejuez CW, Aklin WM, Zvolensky MJ, Pedulla CM (2003) Evaluation of the Balloon Analogue Risk Task (BART) as a predictor of adolescent real-world risk-taking behaviours. Journal of Adolescence 26: 475-479.

Montoya AK, Hayes AF (2016) Two-Condition Within-Participant Statistical Mediation Analysis: A Path-Analytic Framework. Psychological Methods 22: 6-27.
